# Supplementary material for: Protocol for a randomized pilot trial of COMPASS, an open‑source, culturally adapted cognitive behavioral therapy program for forcibly displaced Venezuelan adults in Peru
Source: PLoS One. 2026 Apr 24;21(4):e0345837. doi: 10.1371/journal.pone.0345837 (PMC13108876; doi:10.1371/journal.pone.0345837)
Supplement: S2 File — (PDF) [file pone.0345837.s002.pdf]

**Title of Study:** Adaptation of an open source cognitive behavioral treatment protocol designed to improve mental health in forcibly displaced populations
**IRB Number:** H-45280

**RE:** Initial Review Submission Form
**Review Type:** Expedited
**Action:** Approved

**Date of Approval:** 12/12/2024
**Status Check-In Due Date:** 12/11/2027

**Funding Source:** NIH/National Institute of Mental Health (NIMH)(NIH-NIMH)

December 12, 2024

Dear Haley Carroll,

A qualified member of the Institutional Review Board (IRB) has reviewed the above referenced submission and has determined that the study meets the requirements set forth by the IRB and is hereby approved. This submission was approved by the expedited review process in accordance with the policies and procedures of the Human Research Protection Program (http://www.bumc.bu.edu/ohra/hrpp-policies/hrpp-policies-procedures/#10.2.2).

This approval is valid through the expiration or status check-in due date indicated above.

This approval corresponds with the versions of the application and attachments in the electronic system most recently approved as of the date of this letter. The approved version of the attached protocol is Protocol V.1.0

**Protocol Specific Determinations and Findings**

No PHI collected, accessed, used or distributed under HIPAA per 45 CFR 164.514
Written consent in accordance with 45 CFR 46.117
Inclusion of pregnant persons approved per 45 CFR 46.204

**Resources**
The Clinical Research Resources Office (CRRO) has a number of tools, templated documents, and guidance available for the research community to use. These resources are highly recommended to help

teams meet protocol adherence and regulatory standards. Please review the available resources at the CRRO Resource Library.

**As a principal investigator, you are reminded that you must comply with the responsibilities listed here <http://www.bumc.bu.edu/irb/maintaining-irb-approval/responsibilities-of-the-principal-investigator/>.**

Sincerely,

Lin Themelis, MA, CIP, IRB Administrator

# Adaptation and assessment of a common element treatment protocol designed to improve mental health in forcibly displaced populations

**Protocol Version Number:** 1
**Protocol Version Date:** 25, September 2024
**ClinicalTrials.gov number:** "Pending"
**Funding Mechanism:** National Institute of Mental Health 1K23MH127308

**Principal Investigator:** Haley Carroll
**Phone:** 253-970-0404
**E-mail:** hcarrol1@bu.edu

**CONFIDENTIAL**

**This document is confidential and the property of Boston Medical Center. No part of it may be transmitted, reproduced, published, or used by other persons without prior written authorization from the institution.** © 2024 **Boston Medical Center Corporation. All rights reserved.**

**Summary of Changes:**

| Version | Date | Description of Change | Brief Rationale |
|---|---|---|---|
| 1 | 10/31/2024 | Original Submission | |
| | | | |
| | | | |
| | | | |
| | | | |
| | | | |
| | | | |
| | | | |
| | | | |
| | | | |
| | | | |
| | | | |

## TABLE OF CONTENTS


1 List of Abbreviations ....... 3
2 Protocol Summary ....... 3
3 Background/Rationale & Purpose ....... 5
3.1 Background Information ....... 5
3.2 Rationale and Purpose ....... 6
4 Objectives ....... 7
4.1 Study Objectives ....... 7
4.2 Study Outcome Measures ....... 7
4.2.1 Primary Outcome Measures ....... 7
4.2.2 Secondary Outcome Measures ....... 7
4.2.3 Exploratory Outcome Measures ....... 8
5 Study Design ....... 8
6 Potential Risks and Benefits ....... 9
6.1 Risks ....... 9
6.2 Potential Benefits ....... 10
6.3 Analysis of Risks in Relation to Benefits ....... 10
7 Study Subject Selection ....... 11
7.1 Subject Inclusion Criteria ....... 11
7.2 Subject Exclusion Criteria ....... 11
8 Study Intervention ....... 11
9 Recruitment and Retention Procedures ....... 15
9.1.1 Recruitment Procedures ....... 15
9.1.2 Retention Procedures ....... 16
10 Screening Procedures ....... 16
11 Consent Procedures ....... 17
12 Study Procedures ....... 18
13 Assessment of Safety and Data Safety Monitoring Plan (DSMP) ....... 20
13.1 Definitions for Safety Assessment ....... 20
13.2 Safety Review ....... 21
13.2.1 Multi-Site Safety Monitoring ....... 22
13.3 Reporting Plans ....... 23
13.4 Stopping Rules ....... 23
14 Data Handling and Record Keeping ....... 23
14.1 Confidentiality ....... 23
14.2 Study Documentation, Source Data, and Case Report Forms (CRFs) ....... 24
14.3 Study Records Retention ....... 25
15 Statistical Plan ....... 25
15.1 Study Hypotheses ....... 25
15.2 Sample Size Determination ....... 25
15.3 Statistical Methods ....... 25
16 Ethics/Protection of Human Subjects ....... 26
17 Literature References ....... 26
18 Appendix ....... 28

# 1 List of Abbreviations

| Abbreviation | Definition |
|---|---|
| CMD | Common Mental health Disorder |
| PTSD | Post Traumatic Stress Disorder |
| CBT | Cognitive Behavioral Therapy |
| PHQ9 | Patient Health Questionnaire 9 |
| GAD7 | Generalized Anxiety Disorder 7 |
| LEC5 | Life Events Checklist 5 |
| PCL5 | PTSD Checklist Civilian version 5 |
| IPL | Immigration Policy Lab Integration Index |

# 2 Protocol Summary

| | |
|---|---|
| **Title:** | Adaptation and assessment of a common element treatment approach designed to improve mental health in forcibly displaced individuals. |
| **Population:** | Venezuelan migrants living in Lima (Peru), n = 90 (n = 60 in the intervention and n = 30 in waitlist control), of male and female biological sex, 18 years old or older, with depression (<= 10 in PHQ9), anxiety (<= 10 in GAD7) and/or post-traumatic stress disorder (history of trauma exposure, LEC 5 and <=31 in PCL 5). Participants with suicidality, homicidality or psychosis will be excluded.<br><br>Lay-providers n = 10, of male and female biological sex, 18 years old or older, of any nationality. |
| **Intervention:** | This study is a pilot trial of a culturally adapted transdiagnostic cognitive-behavioral (CBT) intervention for depression, anxiety and post-traumatic stress disorder. The intervention is culturally informed and takes into consideration relevant expectations and practices of Venezuelan migrants living in Lima. It has a duration of 6-12 sessions, depending on the symptom presentation of the participant. The sessions last 60 minutes with a frequency of once session per week. They will be delivered remotely and individually via Meet or a similar platform. The methods employed during the intervention are cognitive re-structuring, behavioral activation, exposure to memories and situations, problem solving, and emotional regulation. The providers will choose from these methods depending on the symptoms found in participants, which they will learn to identify based on assessment information, clinical presentations and discussions with their supervisors and peers. Lay providers will be trained following the apprenticeship model, which is staggered and begins with an active in-person training. In this training strong lay providers |

| | |
|---|---|
| | will be identified and trained as supervisors. Following the training, these supervisors lead small practice groups and supervision groups, closely supported and supervised by psychologists. We will engage a total of n = 10 providers, each of which will engage with n = 9 participants (n = 6 pilot, n = 3 waitlist control). |
| **Objectives:** | To assess the efficacy of an evidence-based, cognitive-behavioral, culturally-adapted, lay-provided intervention on mental health outcomes of adult Venezuelan migrants living in Lima.<br><br>To assess the feasibility and acceptability of the intervention.<br><br>To assess the moderating effect of experiences in migration, integration and demographic variables on the intervention efficacy. |
| **Design/Methodology:** | Migrants will be randomized to receive the intervention (n = 60) or wait list control (n = 30). Mental health outcomes (PHQ9, GAD7, PCL5) will be assessed at baseline during screening, throughout the intervention, post treatment, and at 1-, 3-, and 6- months follow-up. Demographic information, the IPL will be assessed at the time of enrollment. LEC5 will be assessed at baseline and at 1-, 3- and 6-month follow-up. Acceptability will be assessed after each session. All assessments will be conducted by the research team through a secure link in REDCap. Each migrant will participate in 5 assessment sessions and 6-12 intervention sessions with prior assessment of mental health outcomes. The intervention sessions have a frequency of once a week. In the case of the waitlist control, mental health outcomes will be assessed at baseline at the time of enrollment and at endline 12 weeks after baseline. Once the endline has been assessed, they will receive the intervention, with corresponding assessments as described previously. In the case of the providers, feasibility and acceptability will be assessed after each session. See Appendix for a graph of the design, schedule and assessments. |
| **Total Study Duration:** | We expect the first participant to be enrolled in January 2025 and to complete data analysis by April 2027. |
| **Subject Participation Duration:** | Enrollment and initial assessment: 1 online assessment of 30 min<br>Intervention: 6 to 12 online sessions of 60 min in 6to 12 consecutive weeks. A link to assessments will be sent before each session.<br>Post treatment assessment: 1 online assessment of 30 min to be completed within 14 days after completion of the intervention.<br>1-, 3-, and 6-month follow-up assessment: 3 online assessments of 30 min scheduled at 1-, 3-, and 6-months after completion of the intervention. |

# 3 Background/Rationale & Purpose

## 3.1 Background Information

This study aims to address common mental health disorders, such as anxiety, depression and PTSD, among forcibly displaced individuals from Venezuela living in Lima, Peru. To this end a transdiagnostic, cognitive-behavioral, lay-provided intervention will be tested for efficacy, feasibility and acceptability.

Currently there are over 1.5 million Venezuelans living in Peru[1]. Most of them belong to the third wave of migration[2], which began in 2015 as the humanitarian crisis in Venezuela got out of control with extreme inflation rates, severe food and medicine shortages and escalating political and social violence[3]. Evidence suggests that there is a significant need for mental health support in this population. The prevalence of common mental health disorders varies across studies, ranging from a 4.2% of Venezuelan migrants living in Lima with clinically significant symptoms in the Outcome Questionnaire 45.2[4] to 47% of Venezuelan migrants living in Lima and Tumbes reporting 9 to 18 symptoms in the Self Reporting Questionnaire[5]. A study conducted in 2019 in the city of Tumbes in Peru's northern border found that 19% of Venezuelans who were in their migration journey met the criteria for a diagnosis of a provisional depression using the PHQ9 and that 23% qualified for a provisional generalized anxiety disorder in the GAD7[6]. Being a woman, being pregnant during the journey and being discriminated against have been identified as factors that increase the risk of developing symptoms[5–7]. In terms of resources to deal with mental health problems, studies have identified that shelters, which are sometimes the first place where migrants stay upon arrival, frequently provide psychosocial support, even if this is not their primary goal and even when they lack the strategies to do so[8]. Furthermore, community leaders have been found to play a role in establishing contact between the migrants in need of mental health and NGOs offering psychosocial support[5]. With respect to the migrants' access to the formal Peruvian health system, according to the Migration Law, the access to health services is a right of the migrants living in the country no matter their migratory status and it should be guaranteed by the Peruvian State through the Ministry of Health[9]. However, there is no general regulation that clearly establishes the processes involved and, in practice, a foreigner or immigration card, which most Venezuelan migrants living in Peru don't have, is required to affiliate to the insurance and access the services offered by the Ministry of Health[9]. Beyond the regulatory pitfalls it is important to mention that the Peruvian mental health system is characterized by grave deficiencies and inequalities, even for Peruvian citizens[10,11]. The strengthening of the primary and secondary level of attention has been the main goal of a mental health reform under way since 2007, which seeks to build capacities for the detection an initial treatment of mental health disorders in primary health centers and general hospitals and also to build a net of community mental health centers across the country for the provision of out-patient services[11,12].

This study expects to benefit the population of Venezuelan migrants living in Lima, who are suffering from common mental health disorders and have limited access to mental health services. Furthermore, we expect to add to the knowledge regarding CMD lay-oriented interventions for forcibly displaced populations in contexts of resource-scarcity. In terms of the risks, some of questions addressed in the assessments or activities proposed in the sessions might cause discomfort in the participants. Also, as with any mental health intervention, there is always the risk of symptom worsening. The weekly assessment of symptoms throughout the intervention will allow us to closely monitor any changes and put in action a safety plan, in case of increased severity or suicidality.

This study is important because, if proven efficacious, feasible and accepted by the participants, it would constitute an important resource for community leaders, shelter workers, non-governmental organizations and primary level workforce in the provision of mental healthcare specifically tailored for Venezuelan migrants. To date we know of no intervention that has proven its efficacy in the treatment of anxiety, depression and PTSD, that is lay-provided and that takes into consideration the relevant cultural traits and specific needs of the population of Venezuelan migrants living in Lima. It is important to mention that before and during the adaptation process interviews and focus groups were conducted with Venezuelan migrants, experts providing mental healthcare to Venezuelan migrants and experts working in the Peruvian mental health system to gain insight into common stressors, coping strategies, enabling and impeding factors in seeking help, as well as relevant cultural aspects. This feedback has been incorporated into the CBT intervention which will be assessed in this study.

This study will be conducted in compliance with the protocol, applicable regulatory requirements, and policies and procedures of the Boston Medical Center and BU Medical Campus Human Research Protection Program and the IRB office at the subcontract site the Universidad del Pacifico.

## 3.2 Rationale and Purpose

If we consider the pressing need for mental health support of the Venezuelan population in Peru, the role of community organizations and NGOs in providing mental health services, and the urgency for capacity building in the growing primary and secondary level of attention of the formal mental health system, the importance of identifying best practices and designing culturally and contextually appropriate interventions becomes clear. The purpose of this study is to test the efficacy of such an intervention. Previous phases of this study have gathered information via interviews with migrants and experts on relevant cultural and contextual aspects that need to be taken into consideration, when adapting a mental health intervention for the specific population of Venezuelan migrants living in Peru. Alongside these considerations, CBT strategies to treat CMD, that have proven effective in similar populations and that were implemented by lay providers, have been identified. On this basis, strategies have been selected, adapted and presented to experts working in the provision of mental health services, as well as to migrants. Some modifications have been undertaken based in their feedback, resulting in the intervention to be tested in the study described here. Of note, we know of no study to date which has proven the efficacy of an intervention with the characteristics: a) aimed at treating CMD such as anxiety, depression and PTSD; b) provided by lay providers; and c) specifically tailored for Venezuelan migrants established in Lima. Since there is no standard care provided to Venezuelan migrants suffering from CMD, we have opted for a waitlist control study design. As a hypothesis we expect to see a trend toward less depression, anxiety and PTSD over time in the intervention group. Experiences in migration, integration and demographic variables may moderate the efficacy of the intervention.

# 4 Objectives

## 4.1 Study Objectives

The primary objective of the study is to assess the efficacy of an evidence-based, cognitive-behavioral, culturally adapted, lay-provided intervention on mental health outcomes of adult Venezuelan migrants living in Lima. Furthermore, the primary objective is to assess changes in outcomes in depression, anxiety and PTSD symptoms. We expect to see a trend towards less

depression, anxiety and PTSD over time.

The secondary objective is to assess the feasibility and acceptability of such an intervention. Moreover, the secondary objective is to measure the recruitment rate, retention rate, fidelity and acceptability by providers and participants.

The exploratory objective is to assess the moderating effect of experiences in migration, integration and demographic variables on the efficacy of the intervention. Another exploratory objective is to identify characteristics associated with success of the implementation.

## 4.2 Study Outcome Measures

### 4.2.1 Primary Outcome Measures

Since the intervention is transdiagnostic and addresses several CMD, there will be three primary outcome measures. Changes in anxiety will be measured using the GAD7 scale. This is a 7-item instrument that measures generalized anxiety. Changes in depression will be assessed using the PHQ9 questionnaire, which is a 9-item instrument that measures depression. Both instruments have been chosen because they are short, easy to administer and have been widely used in research. Spanish versions of GAD7 and PHQ9 have been validated[13–16] and used in Venezuelan and Peruvian populations[16–19]. Furthermore, prior studies with this same population conducted by the main researcher have used these instruments[6]. Regarding PTSD, changes will be measured using the PCL5 checklist which has been validated for its use in Spanish-speaking populations[20] and utilized in research projects with similar populations[21]. The LEC5, which identifies potentially traumatic events in the life course of the participants, will be used at baseline and in the follow-up assessments along with the PCL5.

The primary outcomes will be measured at baseline during screening and at endline 12 weeks after baseline in the case of waitlist control. In the intervention group, they will be assessed at baseline during screening, weekly (PCL5) or every two weeks (PHQ9 and GAD7) throughout the intervention before the next session, after the last session of the intervention and at 1-, 3-, and 6-month follow-ups after completion of the intervention. Participants will be sent a secure REDCap link, where they will be asked to answer each item of the instruments.

See appendix with the English and Spanish versions of the instruments.

### 4.2.2 Secondary Outcome Measures

Additionally, the feasibility and acceptability of the intervention will be secondary outcomes. Recruitment rate, reasons for not participating, retention rate, fidelity and acceptability by providers and participants will be measured. Regarding fidelity, the providers will perform an adherence checklist and audio record their sessions. Research staff will assess 20% of the recorded sessions for adherence and competence. Regarding acceptability, providers and participants will complete a participant satisfaction assessment for each session. The following instruments will be used to measure the secondary outcomes:

- Feasibility:
    - Recruitment:
        - % of participants who consent to screening
        - % who are eligible who enroll
        - characteristics of refusers and reasons for refusal

        - % who meet eligibility
        - reasons for ineligibility
        - % ineligible for each criterion
    - Retention:
        - X enrolled who completed all study visits and follow-up assessment
    - Fidelity:
        - Adherence scores in provider checklist
        - Adherence and competence scores (0 to 10) from research staff assessment of audio recorded sessions
- Acceptability:
    - Provider perception (assessed after each session):
        - Perception of satisfaction of the participant in the session's helpfulness, enjoyment and relevance (0 to 10)
        - Plans to use material learned in the future (Yes/No)
    - Participant perception (assessed after each session):
        - Direct participant report on the satisfaction in the session's helpfulness, enjoyment and relevance (0 to 10)
        - Plans to use material learned in the future (Yes/No)

Acceptability measures will be collected after each session via a REDCap secure link-sent to each provider and participant by the research team.

4.2.3 Exploratory Outcome Measures

The moderating effect of experiences in migration, integration and demographic variables on the efficacy of the intervention will be explored. To measure these exploratory outcomes the instrument IPL will be used. This is a 24-item instrument that measures the psychological, economic, social, navigational, linguistic and political integration in migrant groups. Another exploratory objective is to identify characteristics associated with success of the implementation. Therefore, a survey on demographics will be administered. This survey will include questions on age, gender, place of residence, marital status, employment situation, employer, current occupation, highest educational level achieved, year and month of arrival to Perú.

The IPL and the demographics form will be administered via a secure link to REDCap before the first session of the intervention.

5 Study Design

This is a randomized pilot study with waitlist control with the aim to compare trends in outcomes between the intervention and control group. Following the intent-to-treat principle, all randomized subjects will be included in the analyses. The sample size for pilot participants is based on a simple method outlined by Viechtbauer[22], which suggests a sample size of n = 60 will be adequate for problem detection. As no treatment exists for forcibly displaced populations, we will randomize n = 30 to waitlist control and treat all participants. The randomization process to intervention or waitlist control will be carried out by a random number generator. Participant with odd numbers will be assigned to the active treatment group and participants with even numbers to the waitlist control group.

The study population comprises outpatient participants, with symptoms of common mental health disorders such as anxiety, depression and/or PTSD (inclusion criteria are described in detail in section 5), adult (>= 18 years old), male and female, of Venezuelan nationality, that

have arrived in Lima in years 2014 to the present.

There will be a planned variation in the dosage of the intervention, which can comprise 6 to 12 sessions. The exact number of sessions will be implemented according to the presentation of symptoms and difficulties of the participant. For instance, if the participant presents depressive symptoms concurrent with PTSD, then, strategies for the treatment of depression will be included alongside with strategies for the treatment of PTSD and the number of sessions will be higher. Also, some components identified as important for this population, such as problem solving or emotional regulation, could be added to the intervention, if they are identified as relevant for the specific participant. For instance, if - alongside with the CMD symptoms - the participant presents concrete problems that need to be tackled (e.g. precarious housing conditions, or lack of and need of childcare support), then the "problem-solving" component will be included as part of the intervention and the number of sessions will be higher. As described in detail in Section 8 "Study Intervention", the first 6 sessions will be intended to treat the most urgent symptoms, and the following sessions will tackle other aspects relevant to the mental health of the participants.

As to the collection of data for the assessment of study objectives, the participant outcomes will be measured using validated instruments such as the PHQ9, GAD7, LEC5 and PCL5. In the case of the intervention group, all outcomes will be measured in the screening, after the last session and at 1-, 3- and 6-month follow-up. In the case of waitlist control, primary outcomes will be assessed at baseline during screening and at endline 12 weeks after baseline. For both the waitlist control and the intervention group, The PHQ9, GAD7, and PCL5 will be assessed throughout the intervention (which occurs at randomization for the intervention group, and 12 weeks after for the waitlist control group.

Furthermore, the assessment of secondary outcomes, such as acceptability and feasibility, will be measured as presented above, in section 4.

See the Appendix for a schematic of the study design.

# 6 Potential Risks and Benefits

## 6.1 Risks

It is unlikely that participants will be at risk of physical harm as a result of the study participation. The primary risks to participants are breach of confidentiality, fatigue or personal discomfort during the intervention sessions or assessments. Participants may find some of the topics addressed during the sessions or some of the questions asked during assessments to be emotionally upsetting and may experience short-term elevations in negative affect during active intervention sessions. As with any study of participants with mental illness, there is always the risk of symptom worsening. The weekly assessment of symptoms throughout the intervention will allow us to closely monitor any changes in the migrant participants and put in action a safety plan, in case of increased severity or suicidality. Weekly meetings with supervisors, who will be closely accompanied by the research team in weekly group meetings, will enable us to timely identify participants whose symptoms are not progressing as expected and to take timely action. At any point in the study if participants exhibit increasing severity of psychiatric symptoms that warrants a higher level of care, they will be referred to the local hospital. If symptoms require immediate medical management, participants' family members will be contacted and recommended to bring the participant to the hospital emergency room.

Furthermore, participants will be advised to call the hotline 113 to get in touch with counselors from the Peruvian mental health system or the number 106 in case they need an ambulance.

In the case of the provider participants, they may find some of the topics addressed during the sessions to be emotionally upsetting or may experience concern regarding the wellbeing of some participants. As mentioned above, frequent meetings with supervisors, closely accompanied by the research team, will enable us to discuss these concerns, to identify if the symptoms of a participant are not progressing as expected and to take timely action. Also, supervisions will provide with the opportunity to tackle the discomfort that a provider might be experiencing and to find means to alleviate it.

Other measure to be taken to protect the participants against risk are the following:

- Team of experienced investigators, with extensive background in forcible displacement and mental health
- Rigorous training on ethical conduct of human research for all personnel and project-related training on migration related trauma and understanding mental health. Also, specific training on processes aimed at guaranteeing privacy and confidentiality will be included.
- Measures to ensure privacy and confidentiality, such as making sure of the non-participation of others in the sessions, maintaining documents and information in locked secure settings, identifying individual subjects by study number without personal identifiers.
- Offering breaks to alleviate fatigue and distress; reminders of voluntariness of their participation in the study
- Plan for referral in case of risk of harm, severe depression and suicidality
- Re-experiencing trauma is a common concern, but the process of exposure has been demonstrated to be effective in hundreds of clinical trials for PTSD[23]. The training and supervision of the providers will ensure the rationale, structure and skills to help participants appropriately engage with trauma stimuli.

## 6.2 Potential Benefits

Regarding the participants, we expect to see a decrease in symptoms related to depression, anxiety and PTSD over time throughout the intervention. Therefore, the main potential benefit to the subjects is an improvement in their mental health. Moreover, since the intervention contemplates “problem solving” and “emotional regulation” components, foreseeable benefits would be improvements in situations that participants identify as problematic, as well as in their capacity to manage distressful emotions. Additionally, since the intervention tackles specific aspects related to migration, such as “migratory grief”, a potential benefit is an improvement in the psychological integration to the host country.

Furthermore, we expect the study to provide knowledge on the efficacy of an intervention specifically tailored to tackled CMD in the population of Venezuelan migrants. As noted above (section 3), no evidence has been generated for the efficacy or CMD interventions that are culturally acceptable and contextually feasible to alleviate anxiety, depression and PTSD among Venezuelan migrants living in Peru, which has received upward 1.5 million migrants from Venezuela. More broadly, we expect the proposed study to provide knowledge about the feasibility and efficacy of a transdiagnostic treatment protocol to target mental health disorders in forcibly displaced populations who have migrated to resource-limited settings. The global prevalence of forcible displacement is estimated to be 70 million per year. While it is difficult to accurately assess the exact burden of disease found in forcibly displaced populations,

calculations of mental health prevalence range from approximately 10-30% for depression, anxiety and PTSD.

In the case of the provider participants, we expect that the training leads to the development of skills that will be of help for them in future opportunities.

### 6.3 Analysis of Risks in Relation to Benefits

The population to which the study subjects belong has very limited access to mental health services (see also section 3). Due to bureaucratic barriers, xenophobic attitudes and a lack of resources, Venezuelan migrants are mostly left out of the mental health system provided by the Peruvian State. Furthermore, decreasing availability of funding is jeopardizing the continuity of mental health services provided by NGOs that prioritize migration, as has been informed to us in focus groups with experts. Therefore, mental health interventions specifically tailored for this population that are lay-oriented and strengthen the resources at community and primary care level are promising. With this study we expect to contribute to expand the mental health services provided to Venezuelan migrants in Peru and to put forward a model that could benefit other populations of migrants or forcibly displaced individuals. Furthermore, in relation to the study participants, we expect to provide an intervention that addresses and alleviates their distress in a context, where it would otherwise be left unattended, due to the restrictions mentioned above. Though distress might increase initially, we are basing our intervention in well supported treatment protocols, that have proven their efficacy in numerous studies in different populations[24–31]. Thus, we expect to overall contribute to the well-being of the subjects.

## 7 Study Subject Selection

### 7.1 Subject Inclusion Criteria

One group of study subjects are outpatient individuals, with symptoms of common mental health disorders, such as anxiety, depression and/or PTSD.

In order to be eligible to participate in this study, an individual must meet all of the following criteria:

- have Venezuelan nationality
- have arrived in Lima in years 2014 to the present
- be 18 years of age or older

In addition, in order to be eligible to participate in this study, an individual must meet ONE of the following criteria:

- score 10 points or more at the PHQ9 or
- score 10 points or more at the GAD7 or
- have a history of trauma exposure as listed in the LEC 5 and score 31 points or more in the PCL5

Another group of study participants are lay providers, who are eligible if the 18 years of age or older.

### 7.2 Subject Exclusion Criteria

Participants with suicidality, homicidality and psychosis will be excluded from the study. An individual is excluded from the study if they endorse suicidal or homidical thoughts in the past

three months, or a lifetime psychosis diagnosis:

- Suicidal ideation: “When someone feels as upset as you do, they may have thoughts that life isn’t worth living. What thoughts have you had like this? Have you had these thoughts in the past three months?”
- Homicidal ideation: “When someone feels as upset as you do, they may have thoughts about hurting the person who has upset or hurt them. What thoughts have you had like this? Have you had these thoughts in the past three months?”
- Psychosis: “Do you have a diagnosis of psychosis or schizophrenia?”

If an individual endorses one of these items they will be referred to relevant care options.

## 8 Study Intervention

The intervention in the present study was developed based on CBT techniques that have proven effective in the treatment of CMD. The intervention underwent a process of cultural adaptation following the Ecological Validity Model developed by Bernal et al. (1995)[32], which has been used in numerous adaptations of psychosocial interventions, for instance by Perera et al. (2020 and 2022)[33,34] in the adaptation of Problem Management Plus for its implementation with Venezuelan migrants in Colombia. In the following paragraphs we describe the intervention that resulted from this process. Figure 1 synthesizes its components:

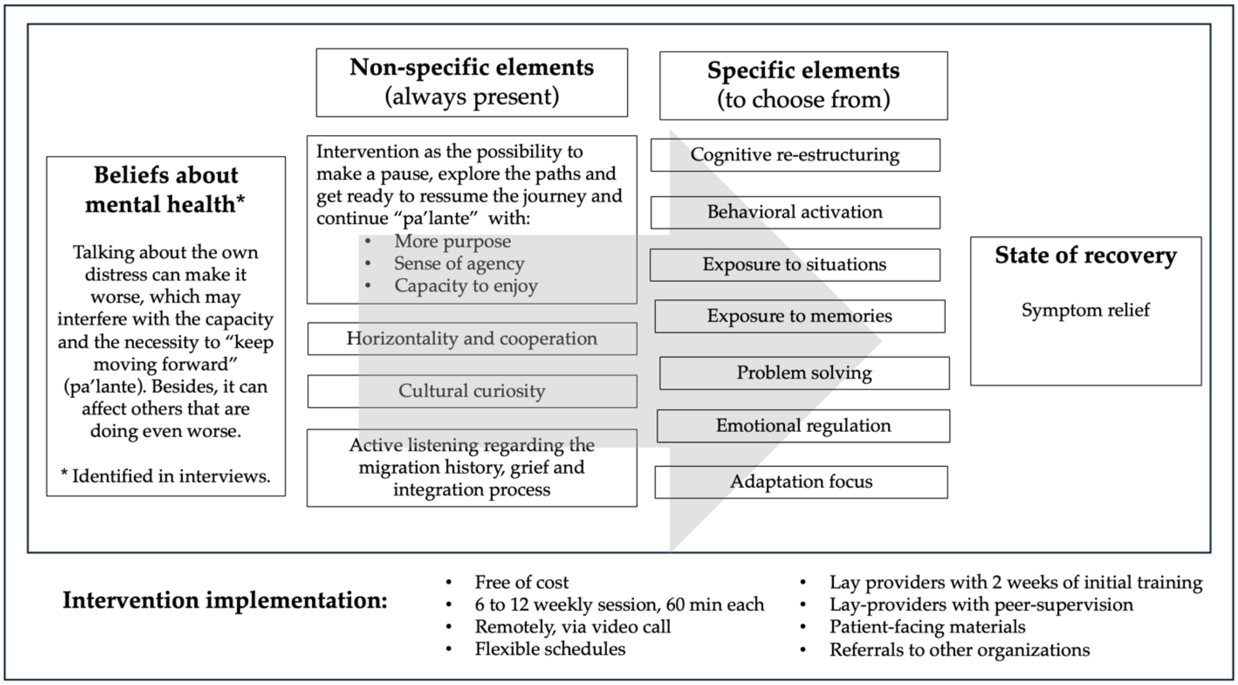


Figure 1. Intervention components and implementation strategies.

In terms of the implementation, the intervention will be free of cost for the participant. It will comprise between 6 and 12 weekly sessions in consecutive weeks and will be delivered remotely via a video call. The sessions will last 60 minutes and before each session participants will be sent a link to REDCap to fill out the questionnaires for the measurement of the primary outcomes. After the session they will answer questions on the session’s acceptability. Since it has been identified that Venezuelan migrants have long working days and might also work in the weekends, we will offer many possibilities to schedule the sessions (e.g. Sundays, early in the morning, late at night…). There will be some flexibility will sessions cancellations and re-scheduling. However, at the beginning of the intervention some rules in this regard will be

agreed upon between provider and participant. A general rule will be set in place and participants will be told that if they can re-schedule a maximum of three sessions in a row and of six sessions overall throughout the intervention. Otherwise, they will be excluded from the study

Regarding the providers, they will receive an intensive in-person two-week training followed by weekly supervisions with their peers. Following the apprenticeship model, 2-3 providers who are identified as “very competent” in the training will assume the role of lay provider supervisors. Lay provider supervisors will be closely supervised by professionally trained counselors (research staff, the US equivalent of bachelors level psychology training or higher) who will in turn be supervised by the primary investigator. Supervision will take place weekly. Competence of providers, lay person supervisors, and supervisors will take place continuously during supervision and be addressed via supervision feedback. This will provide with opportunities to assess any risks and the need to put into action the safety plan, but also to make sound decisions regarding the intervention components based on the information shared in the weekly supervisions and to provide timely support in the implementation of the intervention strategies. Furthermore, providers will have a manual describing each session and the strategies. This material has been written and designed, so that both provider and participant can look at it at the same time. A network of organizations active in mental health and migration will be set in place for providers to make referrals, both as part of the strategies (e.g. a list of organizations that offer community activities for the strategy “behavioral activation”) as well as in case of increasing severity or suicidality, homicidally or psychosis (e.g. community mental health centers in the participant’s neighborhood, organizations that provide support to the victims of domestic violence).

In the intervention itself we have identified via prior interviews that there is a prevalent belief that talking about distress can make it worse and interfere with the possibility of “keep going” to make a daily living, fulfill family duties and accomplish the goals that Venezuelan migrants have set for themselves. Taking this belief into consideration, the intervention begins with an **orientation session**, in which the purpose of the intervention is communicated in terms that make sense for the participant, feedback is provided from the initial assessment and the migratory experience, current difficulties and goals of the participant are explored empathically. Accordingly, the relevant components of the intervention will be briefly presented to the participant, agreed upon and calendarized. This aims to foster an atmosphere of warmth, horizontality and cooperation, which Venezuelan migrants have indicated in interviews is culturally expected.

The **next 3 to 7 sessions** will then tackle the most urgent symptoms identified and discussed with the participants. In an exercise of hope building, the therapist will communicate to the participant that the components presented have shown efficacy in providing relief in people with similar distress. The following components will be suggested in this phase of the intervention:

| **Component** | **Suggested if** |
|---|---|
| Cognitive re-structuring | Always |
| Behavioral activation | PHQ9 => 10 points |
| Exposure to situations | GAD7 => 10 points |

| | |
|---|---|
| Exposure to memories | PCL5 => 31 points |

Here a dosage of the intervention occurs according to the present symptoms. In the case of participants that score higher that the cut-off in all three of the questionnaires applied, more sessions will be dedicated to strategies that tackled symptoms. For participants with a less complex symptomatology, less sessions will be dedicated to this. Once the strategies above have been implemented, participants can opt to continue with **a supplementary set of sessions** (to reach a total of 12 maximum) that may comprise the following components:

| **Component** | **Suggested if** |
|---|---|
| Emotional regulation | Participant describes difficulties identifying, managing and expression emotions (e.g. sudden outbursts of rage, emotional build-up and sudden crying, impulsivity) |
| Problem solving | Participant describes concrete circumstances that generate stress and feels urgent to change (e.g. unemployment, poor housing conditions, lack of support with childcare, etc) and mention feeling stuck or paralyzed regarding their problems, not being able to make a plan, feeling overwhelmed by decisions they should be making. |
| Adaptation focus | Participant describes difficulties in feeling “at home” in the host country (e.g. feeling “out of place”, fear of losing the Venezuelan identity, distrusting and avoiding Peruvians, etc). |

In the following table we will briefly describe each of the components. Each component comes with a “homework” to put into practice in the time between sessions:

| **Component** | **Brief description** |
|---|---|
| Cognitive re-structuring | This is a strategy used in the treatment of different mental disorders. It introduces the triangle “thought-emotion-action” and explores how these three aspects influence each other. Participants examine how a thought can impact the way they feel and their actions. They learn how to “opt” for more helpful thoughts. |
| Behavioral activation | This is a strategy commonly used in the treatment of depression. It introduces the idea that action can precede motivation and not (only) the other way around. Participants are invited to engage in pleasurable activities, which are disaggregated in small and feasible steps. |
| Exposure to situations | This is a strategy used in the treatment of different forms of anxiety. Participants learn that anxiety often leads to avoidant behaviors that exacerbate the distress in a spiral of isolation. By means of facing the dreaded (but harmless situations) gradually, they learn that they can overcome anxious feelings and are able to engage in more activities. |

| | |
|---|---|
| Exposure to memories | This is a strategy used in the treatment of PTSD. There is often concern that intentionally approaching memories that trigger distress will exacerbate symptoms. Participants learn that the avoidance of these triggers is likely what maintains the intrusive memories, which is harmful. They intentionally evoke the memories that provoke the distress and re-learn that these are not dangerous, that they can overcome the distress, and that they will experience less distress over time. |
| Emotional regulation | Participants learn to identify, name and communicate uncomfortable emotions. They also examine their coping strategies, when feeling emotionally overwhelmed, critically assess them in terms of their advantages and disadvantages and expand their repertoire. |
| Problem solving | Participants learn a method to solve problems. They list their most urgent problems, choose one, describe it in detail, gather solutions, select the most feasible strategies and put together an action plan. |
| Adaptation focus | Participants focus on feelings related to having migrated to put into action the strategies mentioned above, but this time with an emphasis on supporting their adaptation to the host country. For instance, participants use the strategy “cognitive re-structuring” to examine the way they feel about having migrated, identify common thoughts related to this and practice new and more helpful ways to think about the place they now live in. Or participants use “behavioral activation” to put into action small changes that might enhance the pleasurable feeling of “being at home” in their “new homes”. Or participants plan an activity with Peruvians (e.g. colleagues, neighbors, parents of their children’s friends), if they tend to avoid hanging out with Peruvians (exposure to situations). Or participants remember the most painful or distressful parts of the experience of having migrated like separating from their children, facing difficulties during the journey or upon arrival (exposure to memories). In terms of emotional regulation, they learn to oscillate between the grief-like feelings and past-focused cognitions and the implementation of healthy coping strategies and present- and future-oriented thinking. |

The **last session** allocates time to review the process and to make a plan to continue to put into practice the strategies learned. It also includes a discussion of possibility of relapse and strategies to prevent it or recover from it.

# 9 Recruitment and Retention Procedures

## 9.1.1 Recruitment Procedures

We will implement opt-in and opt-out recruitment procedures. To do so, we will utilize the support of several institutions: NGOs active in migration, health centers and community mental health centers from different areas of the city, shelters that receive migrants, schools. With their agreement we will carry out the following opt-in recruitment activities:

- Posters will be posted in the front of these organizations describing the study
- Flyers will be made available at these institutions
- Flyers will be handed out at community events organized by NGOs active in migration
- Posts will be published in the social networks of NGOs active in migration

Furthermore, in coordination with NGOs that offer mental health services to Venezuelan migrants, we will ask them to identify potential eligible subject, who - due to a lack of resources - could not be offered support by the NGO. We will ask these organizations to directly reach out to these individuals to provide information about the study and give them the opportunity to learn more by a follow-up call by the research team or not (opt-out procedure). In the follow-up by the research team, individuals are explained about the study. If the person is interested in participating, the screening consent discussion will be conducted followed by the screening procedure. Eventually, we will ask participants to hand-out flyers to acquaintances that could be interested in hearing about the study and let them know that they can contact the research team directly. The script for the recruitment call can be found in the Appendix.
The posters, flyers, publications and verbal communications (e.g. the script for the recruitment call) about the study will include information on the purpose of the study, the eligibility criteria, the commitments, the online modality of the intervention, the contact information. See Appendix.

### 9.1.2 Retention Procedures

This study requires long-term subject participation; therefore, some actions will be undertaken to promote retention. Participants will receive a phone call one day prior to their session as a reminder. If a participant does not connect to the session's link, we will call them 5 minutes after the session time has ended to learn of any unforeseen difficulties and reschedule if necessary. If they don't answer, we will call and text them again the next day and a week after. Also, collateral contact information will be collected to be able to contact them for the 6-month follow-up assessment, in case they have changed phone numbers. Furthermore, to motivate completion, a participant reimbursement (5USD$, apx 20PEN per session attended) in form of a digital giftcard will be sent at the end of the last session via WhatsApp. Lastly, rules regarding rescheduling and no-show will be agreed upon with each participant at the beginning of the intervention. In the preparatory phases of this study, we have learned that Venezuelan migrants living in Lima have long working hours and numerous responsibilities towards their families, both in Lima and in Venezuela. Thus, they have very limited time to invest in any other activity and need a certain degree of flexibility to attend to their sessions. Therefore, we hope that by addressing this at the beginning of the intervention and reaching agreements as to how to proceed, we might promote retention. However, a general rule will be set in place and participants will be told that if they can re-schedule a maximum of three sessions in a row and of six sessions overall throughout the intervention. Otherwise, they will be excluded from the study.

## 10 Screening Procedures

Since the study's main objective is to test the efficacy of an intervention in the treatment of CMD, it is a pre-requisite for participation that individuals present CMD symptoms. Therefore, a screening procedure is necessary. A call will be scheduled with potential participants that contact the research team (opt-in recruitment) or that consent to be contacted by the research

team (opt-out recruitment). In this call the intervention will be described in greater detail (purpose, duration, modality) and the screening consent procedure will be carried out. Potential participants will be informed that:

- This is a research study.
- It is necessary to gather some information to be able to see if the study is suitable for the person.
- A prior requirement is to answer some questions regarding different distressful experiences.
- Based on pilot testing of the assessment, we expect the screening procedure will last no more than 30 min and will be carried out via a telephone call.
- Following completion of the questionnaires the research team will complete a follow up call.
    - Based on responses on the measures/scores, the call will either invite the participant to participate in the research project, or in the case the person is not invited, they will be called and be provided referrals to other organizations that could be able to help them.
- In case the person is invited, they will be randomly assigned to receive treatment nowor in 12 weeks.
- Measures will be taken to guarantee confidentiality and data safety (specific actions will be described).
- They can decide to terminate participation in the screening procedure at any time without any negative consequence to them.
- Their participation in the screening is not tied to their participation in the study. Despite taking part in the screening, they can decide not to participate in the study.

Once this information has been delivered and questions have been clarified, we will collect informed consent for the screening. To this end, participants will receive a secure link to REDCap. Upon consent, participants will be asked questions regarding age, nationality, date of arrival to Peru, suicidality, homicidality, psychosis and the items of the PHQ9, GAD7, LEC5 and PCL5. The researcher will walk the person through the questionnaires during the call, read the questions and mark their answers. If no exclusion and all inclusion criteria apply, individuals will receive a message inviting them to participate in the study. Otherwise, they will be communicated that they cannot be included in the study and information on organizations offering mental health support will be shared with them. If suicidality or homicidality is identified, a safety plan will be implemented. Individuals will be told that their safety is at risk and that we need to contact family member. We will contact a family member and refer to the hospital or other relevant care options, such as the Community Mental Health Centers. Furthermore, individuals will be advised to call the hotline 113 to get in touch with counselors from the Peruvian mental health system.

The Screening Consent Form is attached to this Protocol in a separate document.

## 11 Consent Procedures

Since the participants targeted in this study are over 18 years old, they will give their consent themselves in an electronic informed consent procedure. The process will take place remotely. A call will be scheduled between a member of the research team and the individuals that have been identified as suitable in the screening process.

During the call, a REDCap secure link to the consent form will be sent to the potential

participant and a trained member of the research team will describe the study in non-technical terms in Spanish. Study staff will receive information on the necessary procedures to obtain informed consent, and training and supervision will be repeated to continuously reinforce these procedures. All study staff will also receive training on the ethical conduct of research with human subjects and must recertify annually.

All individuals will be informed that they can withdraw their consent to participate at any time during the study, without any consequences. Potential participants will be informed that the information provided through consent and in the sessions is confidential (i.e., will not be shared with anyone outside the research team) and voluntary (i.e., they are not obligated to share it). The research staff member will emphasize that participation in the study is entirely voluntary, that participants can withdraw their consent and leave the study at any time. The research staff will receive comprehensive training to ensure that participants give their informed consent voluntarily. If at any point the potential participant is not interested in the study, the research staff member will thank them for taking the time to learn about the study and then register the information about why they were not interested in participating.

The informed consent form will provide the following information comprehensively: (a) introduction to the consent process, explaining the consent form and compliance with institutional policy and country laws; (b) reminder that participation is voluntary; (c) nature and purpose of the study; (d) explanation of study procedures; (e) potential discomforts and risks, as well as plans to protect participants from these risks; (f) potential benefits; (g) confidentiality, including how the data will be used and how it will be kept private; (h) refusal/withdrawal, including the right to withdraw consent and leave the study at any time; and (i) rights and complaints. A summary of each section will be read to the participant by the member of the research staff. After each main section, the member of the research team will pause and check comprehension, for example, by asking the participant to repeat, in their own words, what "the right to refuse" means.

Once questions have been clarified and all information has been shared, individuals will be invited to decide whether they want to participate and to give their consent electronically via a link in REDCap. In REDCap they will find the consent form, an affirmation that the understand the study and consent to participate, a box to enter their name and a box to enter the date. Also, they will find two boxes to check, if they give their permission to be contacted again by the research team for the purpose of this study and for other studies. They will be reminded that they can withdraw at any time and referred to the contact information to do so. After the call, participants will be sent a PDF copy of the signed consent form via WhatsApp.

## 12 Study Procedures

See the Appendix for the schedule of events.

| **Phase** | **Time** | **Description** |
| --- | --- | --- |
| Recruitment | Begin: January 2025<br><br>End: When n = 90 is reached (n = 60 intervention; n = 30 waitlist control) | Opt-in: Potential participants learn about the study and contact the research team. Information is shared via<br>• Posters in the front of organizations NGOs active in migration, health centers and community mental health centers, shelters, schools<br>• Flyers available at these institutions |

| | | |
|---|---|---|
| | | • Flyers handed out at community events organized by NGOs active in migration<br>• Posts will be published in the social networks of NGOs active in migration<br>Opt-out: NGOs that offer mental health services to Venezuelan migrants identify potential eligible subject, who - due to a lack of resources - could not be offered support by the NGO. Organizations directly reach out to these individuals to provide information about the study and give them the opportunity to learn more by a follow-up call by the research team or not. |
| Screening Consent | Begin: January 2025 and as potential participants contact research team until n = 90 is reached. | A call will be scheduled to present the information on the screening procedure, clarify doubts and verify comprehension. Electronic consent will be collected via REDCap or DocuSign. |
| Screening/baseline | Begin: January 2025 and as potential participants contact research team until n = | Remotely via REDCap secure link (PHQ9, GAD7, PCL5, LEC5, demographics, exclusion criteria). |
| Participation consent and enrollment | Begin: January 2025 and as potential participants contact research team until n = 90 is reached | A link to the consent form will be sent via WhatsApp. A call will be scheduled to present the information on the study, clarify doubts and verify comprehension. Electronic consent will be collected via REDCap or DocuSign. |
| Randomization and communication on begin of intervention | | Remotely via telephone call<br>Demographics form via REDCap secure link<br>IPL via REDCap secure link |
| Session 1 (Orientation session) | First group of 30 participants:<br>February 2025<br><br>Second group of 30 participants:<br>May 2025<br><br>Third group of 30 participants:<br>August 2025 | Individually with provider<br>Remotely via Meet or WhatsApp videocall<br>60 min duration<br>Focus on getting to know each other, goal-setting, session scheduling, rules for cancellation and re-scheduling. |
| Sessions 2 to 6 | First group of 30 participants:<br>February 2025<br>-May 2025<br><br>Second group of 30 participants: | Individually with provider<br>Remotely via Meet or WhatsApp videocall 60 min duration<br>Focus on symptom relief (depression, anxiety and/or PTSD) |

| | | |
|---|---|---|
| | May-August 2025<br><br>Third group of 30 participants:<br>August -November 2025 | |
| Sessions 7 to 12 | First group of 30 participants:<br>May - August 2025<br><br>Second group of 30 participants:<br>August-November 2025<br><br>Third group of 30 participants:<br>November 2025-February 2026 | Individually with provider<br>Remotely via Meet or WhatsApp videocall<br>60 min duration<br>Focus on other aspects relevant to mental health (emotional regulation, problem solving, adaptation related distress) |
| Assessments throughout intervention | January 2025 to February 2026 | Remotely via REDCap secure link<br>(PCL5 every week before the session, PHQ9 and GAD7 every 2 weeks before the session, acceptability and adherence every week after each session) |
| Endline | May 2025 to February 2026 | Remotely via REDCap secure link (PHQ9, GAD7, LEC5 and PCL5)<br>12 weeks after first session in intervention group (or within 14 days of completion).<br>12 weeks after baseline in waitlist control group. |
| Assessment at 1-month follow-up | August 2025 to January 2026 | Remotely via REDCap secure link (PHQ9, GAD7, LEC5, PCL5) one month after endline (or a maximum of a month later). |
| Assessment at 3-month follow-up | October 2025 to March 2026 | Remotely via REDCap secure link (PHQ9, GAD7, LEC5, PCL5) three months after endline (or a maximum of a month later). |
| Assessment at 6-month follow-up | January to June 2026 | Remotely via REDCap secure link (PHQ9, GAD7, LEC5, PCL5) 6 months after endline (or a maximum of a month later). |
| Data Analysis | June 2026 to July 2027 | Data analysis will take place using data analytic software and be used for publication and dissemination in scholarly journals and conferences. |

## 13 Assessment of Safety and Data Safety Monitoring Plan (DSMP)

### 13.1 Definitions for Safety Assessment

The following definitions will be used in the assessment of safety:

An *Adverse Event (AE)* is any negative outcome that is undesirable and unintended, resulting from a participant's involvement in a study. These events can encompass various forms of harm, including physical, psychological, social, legal, or economic impacts. For instance, in social-behavioral research, an example of an adverse event could be a breach of confidentiality, where a participant's private information is disclosed to individuals not involved in the research team.

An *Unanticipated Problem (UP)* is defined as an adverse event that meets three specific criteria:

1. Unexpected Nature: The adverse event must be unexpected, meaning its nature, severity, or frequency is inconsistent with the known or foreseeable risks described in the research protocol, informed consent documents, or other relevant sources.
2. Relatedness to Research: The adverse event must be related or possibly related to participation in the research. This means there is a reasonable possibility that the event was caused by the research procedures
3. Increased Risk of Harm: The adverse event must suggest that the research places subjects or others at a greater risk of harm than previously known. This assessment involves considering whether the event indicates a new risk that was not anticipated based on prior knowledge of the research and its procedures.

*Serious Adverse Event (SAE)* is any adverse event that

(1) results in death;
(2) is life-threatening;
(3) results in inpatient hospitalization or prolongation of existing hospitalization;
(4) results in a persistent or significant disability/incapacity;
(5) results in a congenital anomaly/birth defect; org abuse).

*Life-threatening* means that the event places the subject at immediate risk of death from the event as it occurred.

*Unanticipated Problem* is defined as an event, experience or outcome that meets **all three** of the following criteria:

- is unexpected; AND
- is related or possibly related to participation in the research; AND
- suggests that the research places subjects or others at a greater risk of harm (including physical, psychological, economic, or social harm) than was previously known or recognized.

*Possibly related* means there is a reasonable possibility that the incident, experience, or outcome may have been caused by the procedures involved in the research

*Unexpected* means the nature, severity, or frequency of the event is not consistent with either:

- the known or foreseeable risk of adverse events associated with the procedures involved in the research that are described in (a) the protocol–related documents, such as the IRB-approved research protocol, any applicable investigator brochure, and the current IRB-approved informed consent document, and (b) other relevant sources of information, such as product labeling and package inserts; or
- the expected natural progression of any underlying disease, disorder, or condition of the subject(s) experiencing the adverse event and the subject's predisposing risk factor profile for the adverse event.

## 13.2 Safety Review

Both the risks listed in Section 4.1 and unknown risks will be monitored as follows:

Information collected as part of this study could reveal symptoms of major depression and suicidal tendencies. All staff will be trained to identify participants who may have acute attacks of anxiety, emotional distress, or suicidal ideation. Throughout the period of interaction with participants, trained staff members will evaluate and be attentive to the emotional state of the participants and lay person therapists. All staff will be trained by the principal investigator in monitoring, evaluating, and reporting any concerns or mental health events. The wellbeing of participants will be assessed in sessions with providers and via validated measures of depression, anxiety, and PTSD provided through the intervention. The wellbeing of lay person therapists will be assessed via supervision sessions with lay person supervisors, study staff supervisors, and the principal investigator Dr. Carroll. All staff must complete human subject research training offered by Boston University Medical Campus and the Collaborative Institutional Training Initiative (CITI Program). All study staff must also complete the Good Clinical Practice training offered within CITI Program. Dr. Carroll has completed Good Clinical Practice Training (Principal Investigator Role: What Every New and Seasoned PI Needs to Know about the Conduct and Oversight of Clinical Research conducted by the Clinical Research Resources Office (CRRO) at Boston University, March 10th, 2023, 3.5 hours), and will complete the refresher course as needed. These extensive and required trainings meet NIH requirements for human subject research training. Dr. Carroll (Principal Investigator of the study, and Licensed Clinical Psychologist) will be available at all times through her direct phone number for questions about participants, as needed, or in case of an emergency. Participants identified as suicidal or severely depressed are provided with information about the most optimal mental health centers or those closest to the participant. In accordance with the Mental Health Law, the confidentiality and voluntariness of going to a mental health center is guaranteed.

If adverse events occur (i.e., events that are harmful to study participants), staff will be trained to complete adverse event descriptions which will then be transmitted electronically to Dr. Carroll within 24 hours. If the event is a UP, it will be reported to the IRB through the electronic IRB system. As this is a multi-site study, the Principal Investigator will report any internal and external UP to both the local IRBs and the primary IRB, following the required guidelines. Reports will be submitted as soon as possible, but no later than 7 days after the investigator or research staff becomes aware of the incident. Each report will describe the UP and specify any required changes to the research or explain why no changes are needed. If changes are necessary, a separate amendment request will also be submitted, but this should not delay the initial report. Additionally, the Principal Investigator will submit a summary report of all AE, including SAE that are NOT classified as UP, to the IRB through the electronic IRB system during the continuing review and at the time of study closure. A formal report from a data safety monitor will fulfill the requirement for this summary report. The summary will include an analysis assessing whether the overall pattern of events indicates that the research poses a greater risk of harm to subjects or others, considering physical, psychological, economic, or social harm based on the nature and frequency of the events. At the time of enrollment, participants will be reminded that the Informed consent form has information on how to contact the research coordinator and the Principal Investigator at the study site to report any adverse events or any questions or concerns related to the study. Expedited review will be conducted for all events that meet the Office for Human Research Participants (OHRP) 2007 definition of Serious Adverse Events (SAEs; that is, any fatal event, immediately life-threatening event, requires inpatient hospitalization, permanently or substantially disabling event, jeopardizes health, or any congenital anomaly). This also includes any event that a study investigator judges to impose a significant hazard, contraindication, side

effect, or precaution. For the purposes of this study, all SAE will be required to report to the BMC IRB (Boston Medical Center) and research mentors, Drs. Feline Freier and Matthew Bird, to the Universidad del Pacífico no matter the judgment regarding their relationship to the study. All pertinent information will be reported, including information about the event and its outcome, concomitant medications, the subject's medical history and current conditions, and all pertinent laboratory data. As noted above, reports of SAE will be submitted as soon as possible, but no later than 7 days after the investigator or research staff becomes aware of the incident. The information will be reviewed, and it will be determined if there was any possible relevance to the study interventions.

If at any time during the study, the investigators, mentors, or IRBs judge that the risk to subjects is greater than the potential benefits, they will have the discretion and responsibility to recommend that the study is terminated. We will use meet the Office for Human Research Participants (OHRP) 2007 definition of Serious Adverse Events (SAEs; that is, any fatal event, immediately life-threatening event, requires inpatient hospitalization, permanently or substantially disabling event, jeopardizes health, or any congenital anomaly).

### 13.2.1 Multi-Site Safety Monitoring

In this multi-site study, we have established a comprehensive plan for the coordination of safety monitoring across all participating sites, as described above. The following summarizes the aforementioned procedures on how communication will be managed regarding information relevant to participant protection, including SAE, UP, protocol modifications, and training and compliance.

1. Regular Communication Protocols
   - Weekly Team Meetings: The PI and research coordinators from each site will participate in regular weekly meetings to discuss ongoing participant safety, review reported events and address any concerns.
   - Monthly Data Review: A systematic review of data will occur monthly, where the research team will evaluate the frequency and nature of reported AEs and SAEs across sites.
2. Inter-Site Communication
   - Immediate Notification of SAEs and UPs: Any site that identifies a SAE or UP will notify the PI and all other sites within 24 hours of awareness. This ensures that all sites remain informed of potential risks and can adjust their monitoring practices accordingly.
   - IRB Reporting: The PI will ensure that all UPs and SAEs are reported to the appropriate Institutional Review Boards (BMC and Universidad del Pacifico IRB) within the required timeframe, with updates communicated to all sites.
3. Protocol Modifications
   - Protocol Amendments: If protocol modifications are necessary based on interim results or safety monitoring, these will be communicated to all sites immediately. Affected sites will receive detailed instructions on implementing changes and will be required to confirm understanding and compliance.
4. Training and Compliance
   - All site staff will undergo training on the safety monitoring procedures and communication protocols established for the study. This training will ensure that every team member understands their role in participant safety and the importance of timely reporting.

By implementing these structured communication and coordination plans, we aim to ensure participant safety across all sites while maintaining compliance with regulatory requirements and ethical standardsReporting Plans

The Principal Investigator at BMC/BU Medical Campus will report Unanticipated Problems, safety monitors' reports, and Adverse Events to the BMC/BU Medical Center IRB in accordance with IRB policies:

- Unanticipated Problems occurring at BMC/BU Medical Campus and the Universidad del Pacífico will be reported to the BMC/BU Medical Campus IRB within 7 days of the investigator learning of the event.
- Adverse Events (including Serious Adverse Events) will be reported in summary at the time of continuing review, along with a statement that the pattern of adverse events, in total, does not suggest that the research places subjects or others at a greater risk of harm than was previously known.
- Reports from safety monitors with recommended changes will be reported to the IRB within 7 days of the investigator receiving the report.
- Reports from safety monitors with no recommended changes will be reported to the IRB at the time of continuing review.

The Principal Investigators at the Universidad del Pacífico will additionally follow the reporting policies and procedures of their local IRB.

### 13.3 Stopping Rules

If at any time during the study, the investigators, mentors, or IRBs judge that the risk to subjects outweighs the potential benefits, they shall have the discretion and responsibility to recommend that the study be terminated. We do not, however, have a pre- specified stopping rule.

## 14 Data Handling and Record Keeping

### 14.1 Confidentiality

As indicated in our research plan, we will do our best to protect participant data in accordance with national confidentiality laws. Participants may be at risk if they reveal a mental disorder in the discussion and their data is leaked (e.g., the social consequences of having a stigmatized disorder). For this reason, we have drawn up strict guidelines on protecting participant confidentiality. All cases of social harm and other adverse events will be brought to the attention of the mentors and the Principal Investigator. Cases of social harm that are determined to be UP, unexpected and serious (or could pose increased risk to subjects), and possibly related to being in the research will be reported to the BMC IRB, the Universidad del Pacífico, and the National Instituted of Health.

Participants will be assigned a unique study ID, with study data associated only with these IDs. Participants will be identified in all study materials solely by participant number, visit number, and visit date. A master list of participants' names and study IDs will be kept in a locked filing cabinet separate from other data. The principal investigator and research study coordinators will be the only individuals with access to this list. The master list will be destroyed at the end of the study. Transcriptions for the assessment of fidelity will use participant numbers, not their names. By recording study data in this manner, the information can be considered "de-identified," thus complying with the Health Insurance Portability and Accountability Act (HIPAA) Privacy Rule standards for individually identifiable health information.

Only the research staff will have access to participants' identifiable private information. No additional contact or data collection will be undertaken for the proposed research. All data is

obtained specifically for research purposes.

All audio recordings, transcripts, notes, and completed qualitative assessments will be stored in a locked filing cabinet and kept in restricted areas. All USB drives and computers will be encrypted and password protected. Electronic versions of audio recordings, transcripts, notes, and quantitative data will be password protected. Passwords will change periodically and will only be accessible to specified research staff. All digital audio recordings will be uploaded to the studio computer immediately after recording and the audio file will be deleted from the digital recorder.

This study will be registered at ClinicalTrials.gov.

## 14.2 Study Documentation, Source Data, and Case Report Forms (CRFs)

Data on eligibility, the participant screening, demographic information, outcome measures (PHQ9, GAD7, LEC5 and PCL5), potential moderator variables (IPL), adherence and acceptability will be entered directly into the electronic system REDCap. Data on feasibility (recruitment and retention) will be entered into an Excel sheet. All the data will be generated by the study procedures and will meet the ALCOA-C criteria.

See Appendix for the forms to be used in REDCap.

Corrections on data collection forms: If any entry error has been made to hardcopy data collection forms, to correct such an error, a single straight line will be drawn through the incorrect entry and the correct data will be entered above it. All such changes will be initialed and dated. There will be no erasures or white-out on CRFs. For clarification of illegible or uncertain entries, the clarification will be printed above the item, then initialed and dated.

See the Appendix for the following CRFs:

Demographics Screening
Psychosis Questions
PHQ9
GAD7
LEC5
PCL5 (monthly)
PCL5 (two-week version)
IPL
Acceptability

Data forms will undergo a systematic and rigorous editing process prior to being keyed into the database. The research coordinator (Talia Guevara, at the subcontract cite Universidad del Pacífico) and PI will routinely evaluate the data and discuss any problems and questions with the investigator team at the regular weekly team meetings. Data management reports across the three following domains will be employed: entered, verified, and edited. These reports of data records will be evaluated once a month. To help ensure data protection, backup copies will be generated each week. Additionally, our hard copy record systems, as described previously, will be maintained in fire-resistant locked cabinets at each site. Data collected in Perú from study assessments, will be will be available for the PI and research staff REDCap. In addition, data on interventionist training, independent assessor training, clinical supervision, and participant progress through the study procedures will also be entered and uploaded. This data entry will occur as close to real time as possible to facilitate data management and monitoring of study operations.

All audio recordings, transcripts, notes and completed quantitative assessments will be stored in a locked file cabinet and kept in restricted areas. All USB flash drives and computers will be password protected and encrypted. Electronic versions of audio recordings, transcripts, notes, and quantitative data will be password protected. Passwords will be changed periodically and only accessible to specified research staff. All digital audio and video recordings will be uploaded to the study computer immediately following recording and the audio file will be deleted from the digital recorder.

Computer files with participant information will be available for PI and research staff at REDCap, a password-protected computer software and website that is compliant with the Health Insurance Portability Act of 1996 (HIPAA) and is used by BMC to transmit confidential clinical and research files. Only the research team, including the PI and the mentoring team, will have access to the data. Recordings will be maintained until seven years after the publication of study results in line with the guidelines of the American Psychiatric Association.

Participants will be assigned a unique study ID, and study data will be associated with participants' study IDs only. Participants will be identified on all study materials only by participant number, visit number, and date of visit. A master list of participant names and their study IDs will be stored in REDCap separate from the rest of the data. The study research coordinators, and the PI will be the only people with access to this list. The master list will be destroyed upon completion of the study. Participants' names will not be used in recorded interviews. By recording the study data in this manner, the information can be considered 'de-identified,' and therefore, compliant with the Standards for Privacy of Individually Identifiable Health Information (Privacy Rule) of HIPAA.

### 14.3 Study Records Retention

Study records, screening consent and participation consent forms and the data collected during the study will be retained for 7 years after the study is closed and then destroyed. They will be preserved in electronic form.

## 15 Statistical Plan

### 15.1 Study Hypotheses

We expect to see a trend toward less depression, anxiety and PTSD over time in the intervention group in comparison to the waitlist control group. Experiences in migration, integration and demographic variables may moderate the efficacy of the intervention.

### 15.2 Sample Size Determination

The sample comprises n = 90 pilot participants, of which n = 60 will be randomly assigned to the intervention group and n = 30 to a waitlist control. The sample size for pilot participants is based on a method outlined by Viechtbauer[22]. Viechtbauer[22] suggests that for a pilot trial a sample size of n = 59 is sufficient to detect a problem that emerges for 5% of participants.

We expect participant recruitment to be feasible as the PI and colleagues at Universidad del Pacifico have recruited and engaged with over 2,000 research participants in mental health research. For example, for the prior stages of this project we have engaged n = 19 experts and n = 29 migrants.

### 15.3 Statistical Methods

All analyses will be performed using multiple packages within SPSS and R. Descriptive statistics will be calculated with frequencies and percentages or means and standard deviations. Demographic variables will be tested for differences in distribution with bi-variate analyses. Variable distributions will be examined, and transformations made, when distributions are skewed or otherwise violate parametric analytic assumptions. All reasons for missing data will be tabulated by reason and reported. All quantitative outcomes will be teste at two-sided $\alpha$=.05 level. Regression analysis will examine changes in primary outcomes in depression, anxiety and post-traumatic stress disorder symptoms and feasibility and acceptability in recruitment rate, assessment completion rate, attendance rate and acceptability rated by providers and participants. Regression will plot the trends in outcome variables over the three study periods (baseline, post-intervention and 6 months following). We will also explore the relationship between covariates and baseline outcome scores and retention, fidelity, and satisfaction to identify any characteristics that might be associated with implementation success. All analyses will adjust for sex as a covariate and it will be tested as an effect modifier (e.g. stratification).

## 16 Ethics/Protection of Human Subjects

This study is to be conducted according to applicable US federal regulations and institutional policies (which are based in federal regulations, guidance, and ICH Good Clinical Practice guidelines).

This protocol and any amendments will be submitted to the Boston Medical Center and Boston University Medical Campus IRB / Universidad del Pacífico for formal approval of the study conduct. The decision of the IRB concerning the conduct of the study will be made in writing to the investigator. A copy of the initial IRB approval letter will be provided to the sponsor, the National Institute of Mental Health, before commencement of this study.

All subjects for this study will be provided a consent form describing this study and providing sufficient information for subjects to make an informed decision about their participation in this study. The consent form will be submitted with the protocol for review and approval by the IRB. The consent of a subject, using the IRB-approved consent form, must be obtained before that subject is submitted to any study procedure. Consent will be documented as required by the IRB.

## 17 Literature References

1. Plataforma Regional de Coordinación Interagencial para Refugiados y Migrantes de Venezuela R4V. R4V América Latina y el Caribe, Refugiados y Migrantes Venezolanos en la Región - May. 2024. Published online May 2024. https://www.r4v.info/es/document/r4v-america-latina-y-el-caribe-refugiados-y-migrantes-venezolanos-en-la-region-may-2024
2. Carhuavilca D, Sánchez A. Condiciones de vida de la población venezolana que reside en el Perú. Resultados de la "Encuesta dirigida a la población venezolana que reside en el país" II ENPOVE 2022. Published online 2022. https://reliefweb.int/report/peru/condiciones-de-vida-de-la-poblacion-venezolana-que-reside-en-el-peru-resultados-de-la-encuesta-dirigida-la-poblacion-venezolana-que-reside-en-el-pais-ii-enpove-2022
3. Freier LF, Parent N. The Regional Response to the Venezuelan Exodus. *Current History*. 2019;118(805):56-61.
4. Delgado-Caceres FM, Silva-Parra KA, Torres-Slimming PA. Association between time of residence and self-perception of distress, interpersonal relationships, and social role in Venezuelan immigrants in Lima, Peru 2018–19: mixed-methods study. *BMC Public Health*.

2022;22. https://doi.org/10.1186/s12889-022-13459-4
5. Centro de Atención Psicosocial. Diagnóstico sobre la situación de salud mental de los refugiados y migrantes venezolanos en Lima y Tumbes. Published online August 2022. https://www.r4v.info/es/document/CAPS_Diagnostico_Salud_Mental_Refugiados_Migrantes_Lima_Tumbes_Ago2022
6. Carroll H, Luzes M, Freier LF, Bird M. The migration journey and mental health: Evidence from Venezuelan forced migration. *SSM Population Health*. 2020;(10). doi:https://doi.org/10.1016/j.ssmph.2020.100551

Mougenot B, Amaya E, Mezones-Holguin E, Rodríguez-Morales AJ, Cabieses B. Immigration, perceived discrimination and mental health: evidence from Venezuelan population living in Peru. *Globalization and Health*. 2021;17(8). https://doi.org/10.1186/s12992-020-00655- 3
7. Blouin C, Jave I. Una mirada a los impactos psicosociales en personas refugiadas y migrantes venezolanas alojadas en albergues en Lima (Perú). Published online November 2019. https://idehpucp.pucp.edu.pe/lista_publicaciones/una-mirada-a-los-impactos-psicosociales-en-personas-refugiadas-y-migrantes-venezolanas-alojadas-en-albergues-en-lima-peru/
8. Blouin C. Estudio sobre el perfil socio económico de la población venezolana y sus comunidades de acogida: una mirada hacia la inclusión. Published online 2019.
9. Rondón M. Peru: Mental Health in a Complex Country. *International Psychiatry*. 2009;6(1). doi:https://doi.org/10.1192/S1749367600000230
10. Tomaya M, Castillo H, Galea JT, et al. Peruvian Mental Health Reform: A Framework for Scaling-up Mental Health Services. *International Journal for Health Policy and Management*. 2017;6(9):501-508. doi:10.15171/ijhpm.2017.07
11. Ministerio de Salud. Decreto Supremo N.° 033-2015-SA. Published online October 5, 2015. https://www.gob.pe/institucion/minsa/normas-legales/193601-033-2015-sa
12. Zhong Q, Gelaye B, Rondón M, et al. Using the Patient Health Questionnaire (PHQ-9) and the Edinburgh Postnatal Depression Scale (EPDS) to assess suicidal ideation among pregnant women in Lima, Peru. *Archives of Women's Mental Health*. 2015;18(6). doi:10.1007/s00737-014-0481-0
13. Zhong Q, Gelaye B, Zaslavsky A, et al. Diagnostic Validity of the Generalized Anxiety Disorder - 7 (GAD-7) among Pregnant Women. *PLoS One*. 2015;10(4). doi:10.1371/journal.pone.0125096
14. García-Campayo J, Zamorano E, Ruiz M, et al. Cultural adaptation into Spanish of the generalized anxiety disorder-7 (GAD-7) scale as a screening tool. *Health and Quality of Life Outcomes*. 2010;8(8). doi:https://doi.org/10.1186/1477-7525-8-8
15. Calderón M, Gálvez-Buccollini JA, Cueva G, Ordoñez C, Bromley C, Fiestas F. Validación de la versión peruana del PHQ-9 para el diagnóstico de depresión. *Revista peruana de medicina experimental y salud pública*. 2012;29(4). doi:https://doi.org/10.1590/S1726-46342012000400027
16. Barrios Y, Gelaye B, Zhong Q, et al. Association of Childhood Physical and Sexual Abuse with Intimate Partner Violence, Poor General Health and Depressive Symptoms among Pregnant Women. *PLoS One*. 2015;10(3). doi:https://doi.org/10.1371/journal.pone.0116609
17. Gomez-Beloz A, Williams MA, Sanchez SE, Lam N. Intimate partner violence and risk for depression among postpartum women in Lima, Peru. *Violence & Victims*. 24(3):380-398.
18. Ghisi GL de M, Santos CVA, Benaim B, et al. Severity of Depressive Symptoms Pre- and Postcardiac Rehabilitation A COMPARISON AMONG PATIENTS IN BRAZIL, CANADA, COLOMBIA, THE UNITED STATES, AND VENEZUELA. *Journal of Cardiopulmonary Rehabilitation and Prevention*. 2017;37(3). doi:10.1097/HCR.0000000000000189
19. Jiménez-Fernández R, Herraiz Soria ME, Peña Granger M, Losa-Iglesias ME, Becerro de Bengoa-Vallejo R, Corral-Liria I. Reliability and validity of the Posttraumatic Stress Disorder

Checklist for DSM-5 (PCL-5) test for post-traumatic stress disorder in mental health nurses in Spain. *Archives of Psychiatric Nursing*. 2024;50. doi:https://doi.org/10.1016/j.apnu.2024.02.006
20. Miller-Suchet L, Camargo N, Sangraula M, et al. Comparing Mediators and Moderators of Mental Health Outcomes from the Implementation of Group Problem Management Plus (PM+) among Venezuelan Refugees and Migrants and Colombian Returnees in Northern Colombia. *International Journal of Environmental Research and Public Health*. 2024;21(5). doi:https://doi.org/10.3390/ijerph21050527
21. Viechtbauer W, Smits L, Kotz D, et al. A simple formula for the calculation of sample size in pilot studies. *Journal of Clinical Epidemiology*. 2015;68(11). doi:10.1016/j.jclinepi.2015.04.014
22. Bradley R, Greene J, Russ E, Dutra L, Westen D. A Multidimensional Meta-Analysis of Psychotherapy for PTSD. *American Journal of Psychiatry*. 162(2). doi:https://doi.org/10.1176/appi.ajp.162.2.214
23. Cuijpers P, Berking M, Dobson KS, Andersson G, Quigley L, Kleiboer A. A Meta-Analysis of Cognitive-Behavioural Therapy for Adult Depression, Alone and in Comparison with other Treatment. *The Canadian Journal of Psychiatry*. 2013;58(7). doi:https://doi.org/10.1177/070674371305800702
24. Cuijpers P, van Straten A, Warmerdam L. Behavioral activation treatments of depression: A meta-analysis. *Clinical Psychology Review*. 2007;27(3):318-326. doi:https://doi.org/10.1016/j.cpr.2006.11.001
25. Bonilla-Escobar F, Fandiño-Losada A, Martínez-Buitrago DM, et al. A randomized controlled trial of a transdiagnostic cognitive-behavioral intervention for Afro-descendants' survivors of systemic violence in Colombia. *PLoS One*. 2018;13(12). doi:doi: 10.1371/journal.pone.0208483
26. Farchione TJ, Fairholme CP, Ellard KK, et al. Unified protocol for transdiagnostic treatment of emotional disorders: a randomized controlled trial. *Behavioral Therapy*. 2012;43(3). doi:10.1016/j.beth.2012.01.001
27. López-López JA, Davies SR, Caldwell DM, et al. The process and delivery of CBT for depression in adults: a systematic review and network meta-analysis. *Psychological Medicine*. 2019;49(12):1937-1947. doi:https://doi.org/10.1017/S003329171900120X
28. Hofmann SG, Smits JAJ. Cognitive-behavioral therapy for adult anxiety disorders: a meta-analysis of randomized placebo-controlled trials. *Journal of Clinical Psychiatry*. 69:621-632.
29. Twomey C, O'Reilly G, Byrne M. Effectiveness of cognitive behavioural therapy for anxiety and depression in primary care: a meta-analysis. *Family Practice*. 2015;32(1):3-15. doi:https://doi.org/10.1093/fampra/cmu060
30. Watts BV, Schnurr PP, Mayo L, Young-Xu Y, Weeks WB, Friedman MJ. Meta-Analysis of the Efficacy of Treatments for Posttraumatic Stress Disorder. *The Journal of Clinical Psychiatry*. 2013;74(6). doi:doi:10.4088/JCP.12r08225
31. Bernal G, Bonilla J, Bellido C. Ecological Validity and Cultural Sensitivity for Outcome Research: Issues for the Cultural Adaptation and Development of Psychosocial Treatments with Hispanics. *Journal of Abnormal Child Psychology*. 1995;23(1). doi:10.1007/BF01447045
32. Perera C, Aldamman K, Hansen M, et al. A brief psychological intervention for improving the mental health of Venezuelan migrants and refugees: A mixed-methods study. *SSM Mental Health*. 2022;2. doi:https://doi.org/10.1016/j.ssmmh.2022.100109
33. Perera C, Salamanca-Sanabria A, Caballero-Bernal J, et al. No implementation without cultural adaptation: a process for culturally adapting low-intensity psychological interventions in humanitarian settings. *Conflict and Health*. 2020;14(46). doi:https://doi.org/10.1186/s13031-020- 00290-0
