# Supplementary material for: Protocol for a randomized pilot trial of COMPASS, an open‑source, culturally adapted cognitive behavioral therapy program for forcibly displaced Venezuelan adults in Peru
Source: PLoS One. 2026 Apr 24;21(4):e0345837. doi: 10.1371/journal.pone.0345837 (PMC13108876; doi:10.1371/journal.pone.0345837)
Supplement: S6 File — (DOCX) [file pone.0345837.s006.docx]

**Metadata**

Funding

The present study is funded by the National Institute of Mental Health (PI: Carroll) 1K23MH127308. The contributions of Dr. Gelaye were made as part of his official duties as a National Institutes of Health (NIH) federal employee, in compliance with agency policy requirements, and are considered Works of the United States Government. However, the findings and conclusions presented in this paper are those of the author(s) and do not necessarily reflect the views of the NIH or the U.S. Department of Health and Human Services.

Competing Interests

The authors have declared that no competing interests exist.

Financial Disclosure

HC; National Institute of Mental Health (PI: Carroll); 1K23MH127308; The sponsor played no role in the study design, data collection and analysis, decision to publish, or preparation of the manuscript. <https://www.nimh.nih.gov/>

The contributions of BG were made as part of his official duties as a National Institutes of Health (NIH) federal employee, in compliance with agency policy requirements, and are considered Works of the United States Government. However, the findings and conclusions presented in this paper are those of the author(s) and do not necessarily reflect the views of the NIH or the U.S. Department of Health and Human Services.

Data Availability

A foundational aspect of COMPASS is its open-sourced nature. Guidance in the foundational phase of this research, including from experts in lay person therapies and the Peruvian mental health system, highlight the cruciality of open sourced, low cost, and flexible therapies for scalability and utility for the Peruvian mental health care system, and eventually for generalizability to other health systems. Thus, the COMPASS manual, training materials, and supervision aides will be made accessible, free of charge following the completion of this study. The clinical outcomes from the study are sensitive, and there is difficult to completely anonymize. To the extent that we can de-identify data we will make it available in accordance to the National Institute of Mental Health and IRB guidance.”

Authors Contributions

HC:
Conceptualization
Data curation
Formal analysis
Funding acquisition
Investigation
Methodology
Project administration
Resources
Software
Supervision
Validation
Visualization
Writing – original draft
Writing – review & editing

TG:
Data curation
Formal analysis
Investigation
Methodology
Project administration
Supervision
Writing – original draft

PG:
Data curation
Formal analysis
Writing – original draft
Writing – review & editing

CM:
Methodology
Supervision
Writing – original draft

SD:
Conceptualization
Funding acquisition
Methodology
Supervision
Writing – original draft
Writing – review & editing

BG:
Conceptualization
Funding acquisition
Supervision
Writing – original draft
Writing – review & editing

DH:
Supervision
Writing – review & editing

MDB:
Conceptualization
Supervision
Writing – original draft
Writing – review & editing

LFF:
Conceptualization
Supervision
Writing – original draft
Writing – review & editing
