# Supplementary material for: Protocol for a randomized pilot trial of COMPASS, an open‑source, culturally adapted cognitive behavioral therapy program for forcibly displaced Venezuelan adults in Peru
Source: PLoS One. 2026 Apr 24;21(4):e0345837. doi: 10.1371/journal.pone.0345837 (PMC13108876; doi:10.1371/journal.pone.0345837)
Supplement: S3 File — (PDF) [file pone.0345837.s003.pdf]

S3 A. Study protocol approved by Universidad del Pacifico in English

UNIVERSIDAD DEL PACÍFICO
Dr. Sánchez Cerro 2141, Jesús María T. (511) 219 0100
Postal Code 15072, Peru – up.edu.pe

No. 008-2025/CIUP/UP

Lima, January 15, 2025

Professor
Matthew Bird
Academic Department of Management
Present.-

Dear Professor,

The purpose of this letter is to greet you and inform you that the Management Committee of CIUP, which serves as the **Research Ethics Committee of the Universidad del Pacífico**, has evaluated and approved the ethical protocols of the following research project under your responsibility:

- **Title:** *"Adaptation and assessment of a common element treatment protocol designed to improve mental health in forcibly displaced populations."*
- **Researchers:** Matthew Bird (Principal Investigator), Haley Ann Carroll, and Luisa Feline Freier.
- **Date of approval by the Committee:** January 14, 2025.
- **Ethical considerations:** The Management Committee of the Research Center of Universidad del Pacífico (CIUP) has approved the ethical procedures proposed for the implementation of screenings, intervention sessions, and interviews that make up the project.

Any changes to the project must be reported to the Committee.

Sincerely,

Gustavo Yamada Fukusaki
Vice Rector for Research

Joanna Kamiche Zegarra
Director
Research Center

Ethical Approval Request Form
CIUP Management Committee – UP Ethics Committee

Instructions :

Below are a series of questions regarding various ethical considerations that your planned research project will require. Please read and answer the questions as clearly and thoroughly as possible. If you have any questions, please do not hesitate to contact the CIUP staff.

I. Study data

i) Project Title

" Adaptation and assessment of a common element treatment protocol designed to improve mental health in forcibly displaced populations" or<br>"Adaptation and evaluation of a Common Elements Treatment protocol designed to improve mental health in forcibly displaced populations."

ii) Professor/Researcher(s) in charge

Haley Ann Carroll
Matthew David Bird Brown
Luisa Feline Freier

iii) The author(s) have the approval of an Ethics Committee external to the University of the Pacific.
( ) Yes. Attach a document that proves this approval and conclude with the required format.
(X) No. Continue. An application has been submitted to the Boston Medical Center Ethics Committee and is currently under review.

iv) Description of the study subjects. Specify whether they are legal entities or natural persons. Detail their characteristics.

One group of study subjects consists of participants with symptoms of common mental health disorders such as anxiety, depression, and/or post-traumatic stress disorder (PTSD). In total, an = 90 participants with these characteristics will be recruited.<br>To be eligible to participate in this study, an individual must meet all of the following criteria:<br>• to have Venezuelan nationality<br>• having arrived in Lima between 2014 and the present<br>• Be 18 years of age or older<br>In addition, to be eligible to participate in this study, a person must meet ONE of the following criteria to be assessed in the screening:<br>• obtain 10 or more points on the PHQ9 [1]or<br>• obtain 10 or more points on the GAD7 [2]or

[1] Patient Health Questionnaire 9
[2] Generalized Anxiety Disorder Questionnaire 7

• have a history of exposure to trauma as indicated in the LEC5 [3]and obtain 31 points or more on the PCL5[4]
A person is excluded from the study if they answer positively to one or more of the following questions during the screening assessment:
• Suicidal thoughts: "When someone feels as upset as you do, they may have thoughts that life is not worth living. What similar thoughts have you had?"
• Homicidal ideation: "When someone feels as upset as you do, they may have thoughts of hurting the person who has upset or hurt them. What similar thoughts have you had?"
• Psychosis: "Do you have a diagnosis of psychosis or schizophrenia?"
If a person confirms one of these points, they will be referred to appropriate care options.

Another group of study subjects consists of participants aged 18 and over who will serve as guides or providers of the intervention. A total of an = 10 participants with these characteristics will be recruited.

v) Description of methods that will be used in the collection of information (interviews, surveys, experiments, etc.).

The primary objective of this study is to evaluate the effectiveness of an evidence-based, culturally adapted, cognitive-behavioral intervention delivered by non-mental health specialists to adult Venezuelan migrants living in Lima. Additionally, the primary objective is to assess changes in depression, anxiety, and PTSD symptoms. We expect to observe a trend toward reduced depression, anxiety, and PTSD over time. The secondary objective is to evaluate the feasibility and acceptability of this intervention. This will be measured by assessing recruitment rates, retention rates, fidelity, and satisfaction levels. The exploratory objective is to evaluate the moderating effect of migration experiences, integration, and demographic variables on the intervention's effectiveness. Another exploratory objective is to identify characteristics associated with successful implementation.

To achieve these objectives, a randomized pilot study with a waitlist control group will be conducted. This will allow for comparison of outcome trends between the intervention and control groups. Following the intention-to-treat principle, all randomized subjects will be included in the analyses. The sample size for the pilot participants is based on a simple method described by Viechtbauer [5], which suggests that a sample size of n = 60 is adequate for problem detection. As there is no standard intervention for forcibly displaced populations, we will randomize n = 30 participants to the waitlist control group and treat all participants. The randomization process for the intervention or waitlist control group will be carried out using a random number generator. Participants with odd numbers will be assigned to the immediate intervention group, and participants with even numbers to the waitlist control group (see the study design diagram in the appendix).

To assess the study objectives, participant outcomes will be measured using validated instruments such as the PHQ-9, GAD-7, LEC-5, and PCL-5 in Spanish. All outcomes will be measured at screening, after the final session, and at 1, 3, and 6

[3] Questionnaire Life Events Checklist 5
[4] Questionnaire PTSD Checklist Civilian version 5
[5] Viechtbauer W, Smits L, Kotz D, et al. A simple formula for the calculation of sample size in pilot studies. *Journal of Clinical Epidemiology* . 2015;68(11). doi:10.1016/j.jclinepi.2015.04.014

months of follow-up. The PHQ-9, GAD-7, and PCL-5 will also be administered throughout the intervention. In addition, implementation fidelity and acceptability will be measured after each session. Feasibility will be assessed at recruitment and retention (see instruments in the appendix).

The intervention in this study was based on cognitive-behavioral therapy techniques, which have proven effective in treating common mental disorders. The intervention underwent a cultural adaptation process following the Ecological Validity Model developed by Bernal et al. (1995) [6], which has been used in numerous adaptations of psychosocial interventions. The main characteristics of the intervention are detailed below.

Regarding implementation, the intervention will be free for participants. It will consist of between 6 and 12 weekly sessions on consecutive weeks and will be delivered remotely via video call. Each session will last 60 minutes, and participants will be sent a secure link to REDCap before each session to complete questionnaires for measuring the main indicators. After each session, they will answer questions about the session's acceptability. Since Venezuelan migrants have been identified as having long working hours and may also work weekends, several scheduling options will be offered for sessions (e.g., Sundays, early morning, late evening, etc.). There will also be some flexibility regarding session cancellations and rescheduling. However, at the beginning of the intervention, some rules regarding this will be agreed upon between the facilitator and the participant. A general rule will be established, and participants will be informed that they may reschedule a maximum of three consecutive sessions and six sessions in total throughout the intervention. Otherwise, they will be excluded from the study.

As for the guides/providers, they will receive intensive two-week in-person training, followed by weekly peer supervision. Following the apprenticeship model, two to three guides identified as highly competent during the training will assume the role of supervisors. They, in turn, will be closely supervised by professionally trained counselors (research staff with a bachelor's degree or higher in psychology) who will, in turn, be supervised by the principal investigator. Supervision will take place weekly. The competence of the providers, non-professional supervisors, and supervisors will be continuously assessed during supervision and addressed through feedback in the supervision sessions. This will provide opportunities to evaluate risks and the need to implement the safety plan. It will also allow for informed decisions regarding the intervention components based on the information shared in the weekly supervision sessions and provide timely support in implementing the intervention strategies. Furthermore, the guides will have a manual describing each session and the strategies. This material will be designed so that both the guide and the participant can consult it simultaneously. A network of organizations active in mental health and migration will be established for guides to make referrals, both as part of the strategies (for example, a list of organizations that offer community activities for the "behavioral activation" strategy) and in case of increased symptom severity or suicidal, homicidal, or psychotic tendencies identified during screening (for example, community mental health centers in the participant's neighborhood, organizations that provide support to victims of domestic violence).

[6] Bernal G, Bonilla J, Bellido C. Ecological Validity and Cultural Sensitivity for Outcome Research: Issues for the Cultural Adaptation and Development of Psychosocial Treatments with Hispanics. *Journal of Abnormal Child Psychology* . 1995;23(1). doi:10.1007/BF01447045

Regarding the sessions, the intervention will begin with an orientation session, in which the purpose of the intervention will be communicated in terms that are meaningful to the participant, feedback on the initial assessment will be provided, and the participant's migration experience, current difficulties, and goals will be explored empathetically. Following this, the relevant components of the intervention will be briefly presented to the participant, the most important ones will be selected, and they will be scheduled. This aims to foster an environment of warmth, equality, and cooperation, which Venezuelan migrants have indicated in interviews as culturally expected.

The following 3 to 7 sessions will address the most pressing symptoms identified and will be discussed with the participants. Offering a sense of hope, the facilitator will communicate to the participant that the presented components have proven effective in providing relief to people with similar distress. The following components will be suggested in this phase of the intervention:

- Cognitive restructuring: always
- Behavioral activation: if the patient has PHQ9 => 10 points
- Exposure to situations: if the patient has GAD7 => 10 points
- Exposure to memories: if the patient has PCL5 => 10 points

The intervention will be adjusted based on the level of distress. Participants who score above the cutoff point on all three questionnaires will receive more sessions focused on symptom-addressing strategies. Participants with less complex symptoms will receive fewer sessions. Once these strategies have been implemented, participants may choose to continue with a supplementary set of sessions (up to a maximum of 12) which may include the following components:

- Emotional regulation: It will be suggested if the participant describes difficulties in identifying, managing and expressing emotions (e.g., outbursts of anger, emotional build-up and sudden crying, impulsivity).
- Problem-solving: It will be suggested if the participant describes specific circumstances that cause them stress and an urgency to change (e.g., unemployment, poor housing conditions, lack of support with childcare, etc.) and if they mention feeling stuck or paralyzed with regard to their problems, unable to generate a plan and overwhelmed by the decisions they should be making.
- Focus on adaptation: It will be suggested, if the participant describes difficulties in feeling “at home” in the host country (for example, feeling “out of place”, fear of losing Venezuelan identity, distrusting and avoiding Peruvians, etc.).

Each session will include an assignment to practice between sessions. The final session will be dedicated to reviewing the process and developing a plan for continuing to implement the learned strategies. It will also include a discussion about the possibility of relapse and strategies for preventing or recovering from it.

The content of each component is briefly described below:

- Cognitive restructuring: This is a strategy used in the treatment of various mental disorders. It introduces the "thought-emotion-action" triangle and explores how these three aspects influence each other. Participants examine how a thought can affect their feelings and actions. They learn to "choose" more helpful thoughts.
- Behavioral activation: This is a strategy commonly used in the treatment of depression. It introduces the idea that action can precede motivation, and not

(only) the other way around. Participants are invited to engage in pleasurable activities, which are broken down into small, achievable steps.

- Exposure to situations: This is a strategy used in the treatment of various forms of anxiety. Participants learn that anxiety often leads to avoidance behaviors that exacerbate it in a spiral of isolation. By gradually confronting feared (but harmless) situations, they learn that they can overcome feelings of anxiety and become more engaged in activities.
- Exposure to memories: This is a strategy used in the treatment of post-traumatic stress disorder. There is often concern that symptoms will be exacerbated if distressing memories are intentionally evoked. Participants learn that avoiding these triggers is likely what keeps the intrusive memories harmful. They intentionally evoke the distressing memories and relearn that they are not dangerous, that they can overcome the distress, and that they will experience less distress over time.
- Emotional regulation: Participants learn to identify, name, and communicate uncomfortable emotions. They also examine their coping strategies when they feel emotionally overwhelmed, critically evaluate them in terms of their advantages and disadvantages, and expand their repertoire.
- Problem-solving: Participants learn a method for solving problems. They list their most pressing problems, choose one, describe it in detail, gather solutions, select the most feasible strategies, and develop an action plan.
- Focus on Adaptation: Participants focus on their migration-related feelings to implement the strategies mentioned above, but this time with an emphasis on supporting their adaptation to the host country. For example, participants use the "cognitive restructuring" strategy to examine how they feel about having migrated, identify common thoughts related to this, and practice new and more helpful ways of thinking about where they now live. Or, participants use "behavioral activation" to implement small changes that could enhance the pleasant feeling of "being at home" in their "new homes." Or, participants plan an activity with Peruvians (e.g., colleagues, neighbors, parents of their children's friends) if they tend to avoid spending time with Peruvians (exposure to situations). Or, participants recall the most painful or distressing parts of their migration experience, such as being separated from their children, facing difficulties during the journey, or upon arrival (exposure to memories). In terms of emotional regulation, they learn to oscillate between grief-like feelings and past-focused thinking, and the implementation of healthy coping strategies and present- and future-oriented thinking.

i. This project is taking place within the framework of some agreement or contract, or possible funding.
   (X) Yes. Describe it

This project is made possible by a grant from the National Institute of Mental Health (Mechanism K23: K23MH127308, https://grants.nih.gov/grants/guide/pa-files/PA-20-206.html, Mentored Patient-Oriented Research Career Development Award) awarded to Dr. Haley Carroll under the mentorship of Drs. Bird and Freier.

   ( ) No.

ii. Indicate probable date of fieldwork: _________January 2025____________

iii. Participant recruitment process
   i) Describe the procedure for recruiting participants. Take into account:

( ) Whether any formal presentation by the University will be required.
( ) Whether any agreement or contract with any institution will be required.
( ) If the study subjects are a vulnerable population (minors, suffer from some disease or special social condition).

For the participant recruitment process, we will rely on the support of several institutions: NGOs active in migration, medical clinics and community mental health centers in different areas of the city, shelters that receive migrants, and schools. With their consent, the following participant recruitment (opt-in) activities will be carried out:

- Posters describing the study will be placed on the facades of these organizations.
- Flyers will be distributed in these institutions
- Flyers will be distributed at community events organized by NGOs active in migration.
- Notices will be published on the social media accounts of NGOs active in migration.

In addition, in coordination with NGOs that provide mental health services to Venezuelan migrants, potential eligible participants will be identified. These individuals, due to a lack of resources, were unable to receive support from the NGOs. These organizations will be asked to contact these individuals directly to provide them with information about the study and offer them the opportunity to learn more through a follow-up call from the research team (opt-out). During the follow-up call, a member of the research team will explain the study. If the individual is interested in participating, a discussion about informed consent for the screening will take place, followed by the screening itself. Participants will then be asked to distribute flyers to acquaintances who might be interested in learning more about the study, informing them that they can contact the research team directly. Posters, flyers, publications, and verbal communications—including the script for the recruitment call—about the study will include information about the purpose of the study, eligibility criteria, commitments, the online modality of the intervention, and contact information (see appendix).

ii) Will there be any payment or other incentive for participation?
(X) Yes. Specify amount and payment procedure:

At the end of the final intervention session, participants will receive USD$5 or PEN 20 per intervention session (6 to 12 sessions) and evaluation session (5 evaluations) they attended. Compensation will be provided for the number of sessions completed, even if participants withdraw from the study before the sessions conclude. This compensation will be in the form of a voucher.

In the case of the facilitators who implement the intervention, they will receive USD$13 or 50 PEN for each session conducted. Payments will be made approximately every two months. They will be compensated for the number of sessions they conduct, even if they withdraw from the study before its completion. In addition, the facilitators will receive compensation for participating in the training, valued at approximately 1500 PEN for 10 days of training.

( ) No.

II. Ensure the voluntary and informed participation of the study subjects (Informed Consent).

i) Specify the procedures that will be used to ensure the voluntary and informed participation of the participants.

Since the primary objective of the study is to test the effectiveness of an intervention in the treatment of common mental disorders (CMD), it is a prerequisite for participation that individuals present with symptoms of this type. Therefore, a screening procedure is necessary, for which a consent procedure is also planned to ensure voluntary and informed participation. To this end, a call will be scheduled with potential participants who contact the research team or who consent to being contacted by the research team. In this call, the intervention will be described in greater detail (objective, duration, modality), and the consent procedure for screening will be carried out. Potential participants will be informed of the following:

- This is a research study.
- It is necessary to gather information in order to determine if the study is suitable for the person.
- A prerequisite is to answer some questions about different experiences of discomfort.
- Based on the pilot tests of the evaluation, we expect the screening procedure to take no more than 30 minutes. It will be conducted via telephone.
- Once the questionnaires are completed, the research team will make a follow-up call. Based on the responses to the measurements/scores, the call will invite the participant to take part in the research project, or if the person is not invited, they will be referred to other organizations that can help them.
- If the person is invited to participate in the study, they will be randomly assigned to receive treatment now or in 12 weeks.
- Measures will be taken to ensure the confidentiality and security of the data (specific actions will be described).
- They can decide to end their participation in the screening procedure at any time without any negative consequences.
- Your participation in the selection process is not linked to your participation in the study. Despite participating in the selection process, you may decide not to participate in the study .

Once this information has been provided and any questions have been answered, we will collect informed consent for the screening using an electronic consent procedure. Participants will receive a secure link to REDCap for this purpose. The informed consent form for the screening is attached.

Participants identified as eligible during screening will receive a message inviting them to participate in the study. Since the target participants for this study are over 18 years of age, they will provide their own informed consent electronically, for which a call will be scheduled. During the call, a trained member of the research team will describe the study in non-technical Spanish. Study staff will receive training on the procedures necessary to obtain informed consent, and this training and supervision will be repeated to continually reinforce these procedures. All study staff will also receive training on ethical conduct in research with human subjects and will be required to recertify annually.

All individuals will be informed that they can withdraw their consent to participate at any time during the study, without any consequences. Potential participants will be informed that the information provided through the consent form and in the sessions is confidential (i.e., it will not be shared with anyone outside the research

team) and voluntary (i.e., they are under no obligation to share it). The research staff member will emphasize that participation in the study is entirely voluntary and that participants can withdraw their consent and leave the study at any time. Research staff will receive comprehensive training to ensure that participants give their informed consent voluntarily. If at any point a potential participant is not interested in the study, the research staff member will thank them for taking the time to learn about the study and will then record the reason why they were not interested in participating.

The informed consent form will provide the following information comprehensively: (a) an introduction to the consent process, explaining the consent form and compliance with institutional policy and the laws of the country; (b) a reminder that participation is voluntary; (c) the nature and purpose of the study; (d) an explanation of the study procedures; (e) potential discomforts and risks, as well as plans to protect participants from these risks; (f) potential benefits; (g) confidentiality, including how the data will be used and kept private; (h) refusal/withdrawal, including the right to withdraw consent and leave the study at any time; and (i) rights and complaints. The research team member will read a summary of each section to the participant. After each main section, the research team member will pause and check for understanding, for example, by asking the participant to repeat, in their own words, what they mean by "the right to refuse."

Once all questions have been answered and all information has been shared, participants will be invited to provide their consent electronically via a link on REDCap . On REDCap, participants will find the consent form, a statement confirming their understanding of the study and their consent to participate, a field to enter their name, and a field to enter the date. They will also find two checkboxes to indicate their permission to be contacted again by the research team regarding this and future studies. Participants will be reminded that they can withdraw at any time and will be provided with the necessary contact information. After the call, participants will receive a signed PDF copy of the consent form via WhatsApp.

Since data will be collected from the guides to measure the feasibility and acceptability of the intervention, they will also undergo an informed consent procedure. The corresponding form is attached.

ii) Attach the Informed Consent form that is planned to be used in the study
Attached are:

- Informed consent form for the screening process
- Informed consent form to participate in the study as a subject
- Informed consent form to participate in the study as a guide.

III. Management of sensitive participant information

i) During the application of the field, personal data will be collected from individuals such as (check those that will be collected):
(X) Names
( ) Initials
(X) Addresses or postal codes
(X) Telephones or emails
(X) Identity document number
( ) Photographs of the participants
(X) Relative data.

( ) None (if you answered this alternative, skip to question iii)

ii) If you are going to collect any data mentioned in the previous question, please indicate whether any procedure will be carried out to anonymize this data.

- Yes, the data will be anonymized. Please describe how this will be done:

Participants will be assigned a unique study ID, and all study data will be associated solely with these IDs. Participants will be identified in all study materials only by their participant number, session number, and session date. A master list of participant names and their study IDs will be kept in a locked filing cabinet, separate from all other data. Only Drs. Carroll, Freier, and Bird, the study's research coordinators, and Talia Guevara, a member of the research team, will have access to this list. The master list will be destroyed upon completion of the study. Participant numbers, not names, will be used in transcripts used to assess the fidelity of implementation. By recording study data in this manner, the information can be considered "non-identified" and therefore complies with HIPAA's Individually Identifiable Health Information Privacy Standards (Privacy Rules) .

- No. Please state why you will not perform this process.

iii) Indicate who will be responsible for safeguarding the collected information.

The person in charge of safeguarding the information and ensuring that it is carried out will be Dr. Haley Carroll, leader of the research team that will carry out this project.

iv) Where will the collected information be stored and will any method be used to safeguard access to it?

Data on eligibility, participant screening, demographic information, outcome measures (PHQ-9, GAD-7, LEC-5, and PCL-5), potential moderating variables (IPL), adherence, and acceptability will be entered directly into the REDCap electronic system, a secure, password-protected, and encrypted software. Data on feasibility (recruitment and retention) will be entered into an Excel spreadsheet.<br><br>Data forms will undergo a systematic and rigorous editing process before being entered into the database. The research coordinator (Talía Guevara, Universidad del Pacífico) and the principal investigator (PI) will routinely review the data and discuss any issues or concerns with the research team during regular weekly meetings. Data management reports will be used in the following three areas: entered, verified, and edited. These data log reports will be reviewed monthly. To help ensure data protection, backups will be generated weekly. In addition, our physical record systems, as previously described, will be kept in fireproof, locked cabinets at each site. Data collected in Peru from study assessments will be available to the PI and research staff at REDCap. Furthermore, data on participant training, supervision, and progress will also be entered and uploaded. This data entry will occur as close to real time as possible to facilitate data management and monitoring of studio operations.

All audio recordings, notes, and completed quantitative assessments will be stored in a locked filing cabinet and kept in restricted areas. All USB drives and computers will be encrypted and password-protected. Electronic versions of the audio recordings, notes, and quantitative data will be password-protected. Passwords will be changed periodically and will only be accessible to designated research staff. All digital audio recordings will be uploaded to the studio computer immediately after recording, and the audio file will be deleted from the digital recorder.

The study records, consent forms for selection and participation, and data collected during the study will be retained for 7 years after the study closes and then destroyed. These will be preserved in electronic format.

IV. Potential risks of the study

i) As part of the study, at some point participants will have to answer questions about sensitive topics (sexuality, politics, etc.):
(X) Yes.
( ) No.

ii) As part of the study, participants will be required to perform some action that may put their physical or mental well-being at risk.
(X) Yes.
( ) No.

iii) As part of the study, the researchers will be required to perform some action that may put their physical or mental well-being at risk.
( ) Yeah.
(X) No.

iv) If you answered “Yes” to any of these questions, describe what these actions consist of and explain the reasons for the need for these actions.

It is unlikely that participants will be at risk of physical harm as a result of participating in the study. The main risks to participants are breaches of confidentiality, fatigue, or personal distress during intervention sessions or assessments. Participants may find some of the topics addressed during sessions or some of the questions asked during assessments emotionally upsetting and may experience short-term increases in negative emotions during intervention sessions. As with any study of participants with psychological distress, there is always a risk that symptoms may worsen. Weekly symptom assessments during the intervention will allow for close monitoring of any changes and the implementation of a safety plan in case of increased severity or suicidal tendencies. Weekly meetings with supervisors, closely supported by the research team in weekly group meetings, will allow for the timely identification of participants whose symptoms are not progressing as expected and for appropriate action to be taken. At any point during the study, if participants exhibit increasing severity of psychiatric symptoms that warrants a higher level of care, they will be referred to the local hospital. If symptoms require immediate medical treatment, participants' families will be contacted and advised to take them to the hospital emergency room. Participants will also be advised to call 113 to contact mental health counselors from the Ministry of Health (MINSA) or 106 if they need an ambulance.

In the case of the participants who guide the intervention, some of the topics discussed during the sessions may be emotionally upsetting, or they may experience concern for the well-being of some participants. As mentioned

previously, frequent meetings with supervisors, closely monitored by the research team, will allow for discussion of these concerns, identification of any participants' symptoms that are not progressing as expected, and timely intervention. Furthermore, supervision will offer an opportunity to address any distress a provider may be experiencing and find ways to alleviate it.

Other measures we will take to protect participants from risks are as follows:

- Team of experienced researchers, with extensive experience in forced displacement and mental health
- Rigorous training in human research ethics for all staff and project-related training on migration-related trauma and mental health understanding.
- Measures to ensure privacy and confidentiality, such as ensuring that no other people participate in the sessions, keeping documents and information in secure and closed environments, identifying individual subjects by study number without personal identifiers
- Offer breaks to relieve fatigue and discomfort; reminders of the voluntary nature of their participation in the study
- Referral plan in case of risk of harm, severe depression and suicidal tendencies
- Re-experiencing trauma is a common concern, but the exposure process has been shown to be effective in hundreds of clinical trials for PTSD [7]. Training and supervision by facilitators will ensure that the rationale is understood, the structure is respected, and the skills are developed to help participants interact appropriately with traumatic stimuli.

That said, it is important to mention that we expect to see a decrease in symptoms related to depression, anxiety, and PTSD over the course of the intervention. Therefore, the main potential benefit for participants is an improvement in their mental health. Furthermore, since the intervention includes components of problem-solving and emotional regulation, the anticipated benefits would be improvements in situations that participants identify as problematic, as well as in their ability to manage stressful emotions. Additionally, given that the intervention addresses specific aspects related to migration, such as migration grief, a potential benefit is improved psychological integration into the host country.

Furthermore, we hope the study will provide insights into the effectiveness of an intervention specifically designed to address common mental disorders in the Venezuelan migrant population. To date, no evidence has been generated on the effectiveness of culturally acceptable and contextually feasible interventions for common mental disorders to alleviate anxiety, depression, and PTSD among Venezuelan migrants living in Peru, which has received more than 1.5 million migrants from Venezuela. More broadly, we hope the proposed study will provide insights into the feasibility and effectiveness of a transdiagnostic treatment protocol to address mental health disorders in forcibly displaced populations who have migrated to resource-limited settings. The global prevalence of forced displacement is estimated at 70 million people per year. While it is difficult to accurately assess the exact burden of disease in forcibly displaced populations, estimates of the prevalence of mental disorders range from approximately 10% to 30% for depression, anxiety, and

[7] Cuijpers P, Berking M, Dobson KS, Andersson G, Quigley L, Kleiboer A. A Meta-Analysis of Cognitive -Behavioural Therapy for Adult Depression, Alone and in Comparison with other Treatment. *The Canadian Journal of Psychiatry* . 2013;58(7). doi:https://doi.org/10.1177/07067437130580070

PTSD .

In the case of the participating providers of the intervention, we hope that the training will contribute to the development of skills that may be useful to them in future opportunities.

The population to which the study participants belong has very limited access to mental health services. Due to bureaucratic barriers, xenophobic attitudes, and a lack of resources, Venezuelan migrants are largely excluded from the mental health system provided by the Peruvian state. Furthermore, the decreasing availability of funds is jeopardizing the continuity of mental health services provided by NGOs that prioritize migration, as reported in focus groups with experts. Therefore, mental health interventions specifically designed for this population, geared toward the general population and strengthening resources at the community and primary care levels, hold promise. With this study, we hope to contribute to expanding mental health coverage. Moreover, with regard to the study participants, we hope to offer an intervention that addresses and alleviates their distress in a context where it would otherwise go unmet due to the aforementioned restrictions. Although discomfort may initially increase, the intervention is based on well-supported treatment protocols that have proven effective in numerous studies across diverse populations. Therefore, it is expected to contribute to the overall well-being of the participants.

V. Fieldwork tools and applications

i) Describe how the fieldwork will be conducted. Indicate whether it will be done in person or online, the intervention timeframe, settings, etc.

Recruitment:

Opt-in: Potential participants are informed about the study and contacted by the research team. Information is shared through

- Posters on the facades of NGOs active in migration, health centers and community mental health centers, shelters, schools
- Flyers are available at these institutions.
- Flyers distributed at community events organized by NGOs active in migration
- Notices will be published on the social media accounts of NGOs active in migration.

Opt-out: NGOs that provide mental health services to Venezuelan migrants identify potentially eligible participants who, due to a lack of resources, the NGO was unable to support. The organizations contact these individuals directly to provide information about the study and offer them the option of receiving further information through a follow-up call from the research team.

Consent for screening:

A call will be scheduled to present information about the evaluation procedure, answer questions, and verify understanding. Electronic consent will be collected through REDCap.

Screening assessment and baseline:

It will be conducted online via a secure link to REDCap. The following questionnaires will be administered: PHQ9, GAD7, PCL5, LEC5, demographic questionnaire, and questionnaire with exclusion criteria.

Consent for participation and enrollment:

A link to the consent form for participation will be sent via WhatsApp to eligible participants. A call will be scheduled to present information about the evaluation

procedure, answer questions, and verify understanding. Electronic consent will be collected through REDCap.

Randomization and communication of the start of the intervention:

The results of the random assignment to "immediate intervention" or "waiting list control group," to be performed by a number generator, will be communicated via telephone call. The completed demographic information form and the IPL questionnaire will be sent via a secure REDCap link.

Session 1 (orientation session):

It will be implemented online via Meet or WhatsApp, individually with the guide. It will last 60 minutes. The focus will be on getting to know each other, setting goals, scheduling sessions, and agreeing on procedures for canceling and postponing appointments.

Sessions 2 to 6:

They will be implemented online via Meet or WhatsApp, individually with the guide. They will last 60 minutes. They will focus on symptom relief (depression, anxiety, post-traumatic stress).

Sessions 7 to 12:

They will be implemented online via Meet or WhatsApp, individually with the guide. They will last 60 minutes. They will focus on other aspects relevant to mental health (emotional regulation, problem-solving, adjustment-related distress).

Assessments during the intervention:

They will be carried out online via a secure link to REDCap (PCL5 every week before each session, PHQ9 and GAD7 every two weeks before the session, acceptability and adherence/fidelity every week after each session).

Final line and follow-up assessments at one month, three months, and six months:

They will be carried out online via a secure link to REDCap (PHQ9, GAD7, LEC5, PCL5) 12 weeks after the first intervention session or 12 weeks after the baseline in the case of the waiting list control group and 1 month, 3 months and 6 months after the final baseline.

Data analysis:

Data analysis will be performed using data analysis software and will be used for publication and dissemination in academic journals and conferences.

Security monitoring and reporting:

An adverse event (AE) is any undesirable and involuntary negative outcome resulting from a subject's participation in a study. These events can encompass various forms of harm, including physical, psychological, social, legal, or economic impacts. For example, in social and behavioral research, an example of an adverse event could be a breach of confidentiality, where a participant's private information is disclosed to individuals not involved in the research team.

Unanticipated Problem (ANP): Defined as an adverse event that meets three specific criteria:

1. Unexpected nature: The adverse event must be unexpected, that is, its nature, severity or frequency must not match the known or foreseeable risks described in the research protocol, informed consent documents or other relevant sources.
2. Relationship to the research: The adverse event must be related or possibly related to participation in the research. This means there is a reasonable possibility that the event was caused by the research procedures.
3. Increased risk of harm: The adverse event should suggest that the research places subjects or others at a greater risk of harm than was previously known. This assessment involves considering whether the event indicates a new risk that was not anticipated based on prior knowledge of the research and its procedures.

Serious Adverse Event (SAE) : any adverse event that
1. It results in death;
2. It is potentially deadly;
3. It results in hospitalization or prolongation of an existing hospitalization;
4. It results in a persistent or significant disability/incapacity;
5. It results in a congenital anomaly/malformation at birth; or abuse.

Potentially fatal means that the event places the subject at immediate risk of death due to the event as it occurred.

The procedures for protecting participants are described below:

1. Regular Communication Protocols
   - Weekly Team Meetings: The principal investigator (PI) and research coordinator will participate in regular weekly meetings to discuss ongoing participant safety, review reported events, and address any concerns.
   - Monthly Data Review: A systematic review of the data will be carried out each month, in which the research team will assess the frequency and nature of reported adverse events and serious adverse events.
2. Inter-Site Communication
   - Immediate Notification of AEGs and PNAs: Any member of the investigation team who identifies a serious adverse event or an unanticipated problem will notify the PI within 24 hours of identifying the event. This ensures that all sites are informed of potential risks and can adjust their monitoring practices accordingly.
   - Report to the IRB: The PI will ensure that all PNAs and EAGs are reported to the appropriate Institutional Review Boards (IRBs) (BMC and Pacific University IRB) within the required timeframe, and updates will be provided to all sites.
3. Amendments to the Protocol
   - Protocol Amendments: If modifications to the protocol are required based on interim results or security monitoring, these will be communicated immediately to all sites. Affected sites will receive detailed instructions on how to implement the changes and will be asked to confirm their understanding and compliance.
4. Training and Compliance
   - Site Staff Training: All site staff will receive training on the security monitoring procedures and communication protocols established for the study. This training will ensure that each team member understands their role in participant safety and the importance of timely reporting.

<u>Reporting plan:</u>

If adverse events occur (i.e., events that are harmful to study participants), staff will be trained to complete adverse event descriptions, which will be transmitted electronically to Dr. Carroll within 24 hours. If the event is an Unanticipated Problem (ANP), it will be reported to the IRB through the BMC (Boston Medical Center) electronic IRB system. Because this is a multisite study, the Principal Investigator (PI) will report any internal and external ANPs to the local IRBs and the main IRB, following the required guidelines. Reports will be submitted as soon as possible, but no later than 7 days after the investigator or research staff become aware of the incident. Each report will describe the ANP and specify any required changes to the research or explain why changes are not necessary. If

changes are required, a separate amendment request will be submitted, but this should not delay the initial report.

In addition, the Principal Investigator will submit a summary report of all Adverse Events (AEs), including Serious Adverse Events (SAEs) that are NOT classified as PNAs, to the IRB via the BMC (Boston Medical Center) IRB electronic system during ongoing review and at study closure. A formal report from a data safety monitor will fulfill the requirement for this summary report. The summary will include an analysis assessing whether the overall pattern of events indicates that the research presents an increased risk of harm to subjects or others, considering physical, psychological, economic, or social harm based on the nature and frequency of the events.

At the time of registration, participants will be reminded that the informed consent form contains information on how to contact the research coordinator and the Principal Investigator at the study site to report any adverse events or any questions or concerns related to the study.

An expedited review will be conducted for all events that meet the Office for Human Participation in Research (OHRP) 2007 definition of Serious Adverse Events (SAEs) (i.e., any fatal event, immediately fatal event, event requiring inpatient hospitalization, event that permanently or substantially disables, event that endangers health, or event that results in congenital anomaly). This also includes any event that a study investigator considers to pose a significant hazard, contraindication, side effect, or precaution.

For the purposes of this study, all adverse events (AEs) must be reported to the Boston Medical Center (BMC) IRB, the research mentors, Drs. Feline Freier and Matthew Bird, and the University of the Pacific, regardless of their relationship to the study. All pertinent information must be reported, including details of the event and its outcome, concomitant medications, the subject's medical history and current conditions, and all relevant laboratory data. As mentioned previously, AE reports These reports will be sent as soon as possible, but no later than 7 days after the investigator or research staff become aware of the incident. The information will be reviewed, and a determination will be made as to whether it has any potential relevance to the study interventions. However, neither the University of the Pacific nor the researchers representing it assume any costs or compensation that may arise. Participation is voluntary and informed (participants may withdraw at any time), and as such, it is not appropriate to assume any costs for treatment, care, counseling, or damages.

<u>Interruption rules:</u>
If at any point during the study the researchers, mentors, or Institutional Review Boards (IRBs) determine that the risk to subjects outweighs the potential benefits, they have the discretion and responsibility to recommend that the study be terminated. However, we do not have a pre-established termination rule.

ii) Please attach the instruments to be used, whether interview protocols, surveys or other materials exactly as they will be presented to the participant.
    Attached (see appendix).

iii) Will recordings or any other type of audio or video recording be made of the participants?

(X) Yes. Indicate what will be used and whether this will be stated in the Informed Consent.

The intervention sessions will be audio-recorded with the participant's permission, following the informed consent procedure. Twenty percent of the session recordings will be selected for fidelity and quality analysis of the intervention. These audio recordings will be transcribed verbatim and translated. An adherence form will be applied to the transcripts (see attached). The results of this evaluation will serve as the basis for group discussion and feedback to the facilitators.

( ) No.

iv) Will any logo or graphic identity of the Universidad del Pacífico be used during the application?

(X) Yes. Describe and attach, if necessary, which ones.

( ) No.

See attached poster template.

VI. Potential conflicts of interest

i) According to the University's Code of Ethics, potential conflicts of interest may arise from the following situations:

- When the researcher or their close relatives have a financial interest in any entity sponsoring the research or have a direct economic interest in the result, or when they can otherwise personally benefit from it.
- When the hiring of close relatives of the researcher is proposed for the project, without them having gone through a transparent selection process.
- When there is a conflict of interest between a researcher's external activities and their responsibilities as a university employee, university professors and researchers must dedicate their time and provide their professional services primarily to this institution.
- When the University can be committed to endorsing or approving any company, commercial product, bill, or partisan stance.
- When the researcher, whose main affiliation is with the University, also has affiliations with entities in the private business sector or the government, and at the same time appears in the media as an expert, without clearly disclosing these affiliations.

ii) If you believe there may be a conflict of interest in your study for the reasons stated above or others, please indicate them below. If there are no conflicts of interest, please also indicate this:

There are no conflicts of interest.

S3 B. Study protocol approved by Universidad del Pacifico in Spanish

[redacted]

Jr. Sánchez Cerro 2141, Jesús María
Código Postal 15072, Perú

T. (511) 219 0100
up.edu.pe

N° 008-2025/CIUP/UP

Lima, 15 de enero de 2025

Profesor
Matthew Bird
Departamento Académico de Gestión
Presente.-

De nuestra especial consideración:

La presente tiene por finalidad saludarlo e informarle que el Comité de Gestión del CIUP, que cumple las veces de Comité de Ética de Investigación de la Universidad del Pacífico, ha evaluado y aprobado los protocolos éticos del siguiente proyecto de investigación que se encuentra a su cargo:

- Título: "Adaptation and assessment of a common element treatment protocol designed to improve mental health in forcibly displaced populations".
- Investigadores: Matthew Bird (Investigador Principal), Haley Ann Carroll y Luisa Feline Freier.
- Fecha de aprobación por Comité: 14 de enero de 2025.
- Consideraciones éticas: el Comité de Gestión del Centro de Investigación de la Universidad del Pacífico (CIUP) ha aprobado los procedimientos éticos planteados para la aplicación de tamizajes, sesiones de intervención y entrevistas que conforman el proyecto.

Cualquier cambio en el proyecto, deberá ser informado al Comité.

Atentamente,

[redacted]

Formato de Solicitud de Aprobación Ética
Comité de Gestión CIUP – Comité de Ética UP

Indicaciones:

A continuación, se presentan una serie de preguntas sobre diferentes consideraciones éticas que deberá tener el proyecto de investigación que planea desarrollar. Por favor, lea y responda las preguntas con el mayor detalle y claridad. Si tuviera alguna consulta, no dude en comunicarse con el personal CIUP.

I. Datos del estudio

i) Título del proyecto

"Adaptation and assessment of a common element treatment protocol designed to improve mental health in forcibly displaced populations" o<br>"Adaptación y evaluación de un protocolo de Tratamiento de Elementos Comunes diseñado para mejorar la salud mental en poblaciones desplazadas por la fuerza."

ii) Profesor/Investigador(es) responsable(s)

Haley Ann Carroll
Matthew David Bird Brown
Luisa Feline Freier

iii) El (los) autor(es) cuentan con la aprobación de algún Comité de Ética externo a la Universidad del Pacífico.
( ) Sí. Adjuntar documento que acredite esta aprobación y concluir con formato.
(X) No. Continuar. Se ha presentado una solicitud al Comité de Ética de Boston Medical Center, que se encuentra en proceso de aprobación.

iv) Descripción de los sujetos de estudio. Especificar si se trata de personas jurídicas o naturales. Detallar las características de estos.

Un grupo de sujetos de estudio está compuesto por participantes con síntomas de trastornos de salud mental comunes como ansiedad, depresión y/o trastorno de estrés post-traumático (TEPT). En total, se reclutará a n = 90 participantes con estas características.<br>Para ser elegible para participar en este estudio, una persona debe cumplir con todos los criterios siguientes:<br>• tener nacionalidad venezolana<br>• haber llegado a Lima en los años 2014 al presente<br>• tener 18 años de edad o más<br>Además, para ser elegible para participar en este estudio, una persona debe cumplir con UNO de los siguientes criterios a evaluarse en el tamizaje:<br>• obtener 10 puntos o más en el PHQ9[1] o<br>• obtener 10 puntos o más en el GAD7[2] o<br>• tener antecedentes de exposición a trauma como se indica en el LEC5[3] y obtener 31 puntos o más en el PCL5[4]

[1] Cuestionario Patient Health Questionnaire 9
[2] Cuestionario Generalized Anxiety Disorder 7
[3] Cuestionario Life Events Checklist 5
[4] Cuestionario PTSD Checklist Civilian version 5

Se excluye a una persona del estudio, si responde positivamente a una o más de las siguientes preguntas durante la evaluación de tamizaje:
• Ideación suicida: "Cuando alguien se siente tan molesto como usted, puede tener pensamientos de que la vida no vale la pena vivirla. ¿Qué pensamientos similares ha tenido?"
• Ideación homicida: "Cuando alguien se siente tan molesto como usted, puede tener pensamientos de lastimar a la persona que lo ha molestado o lastimado. ¿Qué pensamientos similares ha tenido?"
• Psicosis: "¿Tiene un diagnóstico de psicosis o esquizofrenia?"
Si una persona ratifica uno de estos puntos, se la derivará a opciones de atención pertinentes.

Otro grupo de sujetos de estudio está compuesto por participantes de 18 años a más que cumplan la función de guías o proveedores de la intervención. En total se reclutará a n = 10 participantes con estas características.

v) Descripción de métodos que se emplearán en el recojo de información (entrevistas, encuestas, experimentos, etc.).

El objetivo principal del estudio es evaluar la eficacia de una intervención basada en evidencia, cognitivo-conductual, adaptada culturalmente y proporcionada por no-especialistas en la salud mental de los migrantes venezolanos adultos que viven en Lima. Además, el objetivo principal es evaluar los cambios en los valores de depresión, ansiedad y síntomas de TEPT. Esperamos ver una tendencia hacia una menor depresión, ansiedad y TEPT con el tiempo. El objetivo secundario es evaluar la viabilidad y aceptabilidad de dicha intervención. Para esto se medirá la tasa de reclutamiento, la tasa de retención, la fidelidad y el nivel de satisfacción con la intervención. El objetivo exploratorio es evaluar el efecto moderador de las experiencias en migración, integración y variables demográficas sobre la eficacia de la intervención. Otro objetivo exploratorio es identificar las características asociadas con el éxito de la implementación.

Para lograr estos objetivos se realizará un estudio piloto aleatorizado con un grupo de control en lista de espera. Esto permitirá comparar las tendencias en los resultados entre el grupo de intervención y el grupo de control. Siguiendo el principio de "intention to treat", todos los sujetos aleatorizados se incluirán en los análisis. El tamaño de la muestra de los participantes en el piloto se basa en un método simple descrito por Viechtbauer[5], que sugiere que un tamaño de muestra de n = 60 es adecuado para la detección de problemas. Como no existe ninguna intervención estándar para las poblaciones desplazadas por la fuerza, aleatorizaremos n = 30 al grupo control en lista de espera y trataremos a todos los participantes. El proceso de aleatorización para el grupo de intervención o el grupo control en lista de espera se llevará a cabo mediante un generador de números aleatorios. Los participantes con números impares se asignarán al grupo de intervención inmediata y los participantes con números pares al grupo control en lista de espera (ver esquema del diseño del estudio en el apéndice).

Para evaluar los objetivos del estudio, los resultados de los participantes se medirán utilizando instrumentos validados como PHQ9, GAD7, LEC5 y PCL5 en español.

[5] Viechtbauer W, Smits L, Kotz D, et al. A simple formula for the calculation of sample size in pilot studies. *Journal of Clinical Epidemiology*. 2015;68(11). doi:10.1016/j.jclinepi.2015.04.014

Todos los resultados se medirán en la selección, después de la última sesión y en el seguimiento a los 1, 3 y 6 meses. El PHQ9, GAD7 y PCL5 se aplicarán también a lo largo de la intervención. Además, se medirá la fidelidad de la implementación y la aceptabilidad después de cada sesión. Con respecto a la viabilidad, se medirá en el reclutamiento y en la retención (ver instrumentos en el apéndice).

La intervención del presente estudio se desarrolló en base a técnicas de terapia cognitivo-conductual, que han demostrado ser efectivas en el tratamiento de trastornos mentales comunes. La intervención pasó por un proceso de adaptación cultural siguiendo el Modelo de Validez Ecológica desarrollado por Bernal et al. (1995)[6], el cual ha sido utilizado en numerosas adaptaciones de intervenciones psicosociales. A continuación, se detallan las principales características de la intervención.

En cuanto a la implementación, la intervención será gratuita para el participante. Constará de entre 6 y 12 sesiones semanales en semanas consecutivas y se impartirá de forma remota mediante una videollamada. Las sesiones tendrán una duración de 60 minutos y antes de cada sesión se enviará a los participantes un enlace seguro a REDCap para que rellenen los cuestionarios para la medición de los indicadores princilaes. Después de la sesión, responderán preguntas sobre la aceptabilidad de la sesión. Dado que se ha identificado que los migrantes venezolanos tienen jornadas laborales largas y también podrían trabajar los fines de semana, se ofrecerán varias opciones para programar las sesiones (por ejemplo, los domingos, temprano en la mañana, tarde en la noche…). También habrá cierta flexibilidad en cuanto a la cancelación y reprogramación de sesiones. Sin embargo, al inicio de la intervención se acordarán algunas reglas al respecto entre el guía y el participante. Se establecerá una regla general y se les informará a los participantes que podrán reprogramar un máximo de tres sesiones consecutivas y seis sesiones en total durante toda la intervención. De lo contrario, serán excluidos del estudio.

En cuanto a los guías/proveedores, recibirán una formación presencial intensiva de dos semanas de duración, seguida de supervisiones semanales con sus pares. Siguiendo el “apprenticeship model”, 2 a 3 guías, que sean identificados como “muy competentes” en la capacitación, asumirán el papel de supervisores. A su vez, ellos estarán estrechamente supervisados por consejeros profesionalmente capacitados (personal de investigación, con formación en psicología de nivel licenciatura o superior) que, a su vez, estarán supervisados por el investigador principal. La supervisión se llevará a cabo semanalmente. La competencia de los proveedores, supervisores no profesionales y supervisores se evaluará de manera continua durante la supervisión y se abordará a través de retroalimentación en las sesiones de supervisión. Esto brindará oportunidades para evaluar los riesgos y la necesidad de poner en práctica el plan de seguridad. También permitirá tomar decisiones acertadas con respecto a los componentes de la intervención en base a la información compartida en las supervisiones semanales y brindar apoyo oportuno en la implementación de las estrategias de intervención. Además, los guías contarán con un manual que describe cada sesión y las estrategias. Este material estará diseñado para que tanto el guía como el participante puedan consultarlo al mismo tiempo. Se establecerá una red de organizaciones activas en salud mental y

[6] Bernal G, Bonilla J, Bellido C. Ecological Validity and Cultural Sensitivity for Outcome Research: Issues for the Cultural Adaptation and Development of Psychosocial Treatments with Hispanics. *Journal of Abnormal Child Psychology*. 1995;23(1). doi:10.1007/BF01447045

migración para que los guías realicen derivaciones, tanto como parte de las estrategias (por ejemplo, una lista de organizaciones que ofrecen actividades comunitarias para la estrategia "activación conductual") como en caso de aumento de la gravedad de los síntomas o de tendencias suicida, homicida o psicosis, identificadas en el tamizaje (por ejemplo, centros de salud mental comunitarios en el vecindario del participante, organizaciones que brindan apoyo a las víctimas de violencia doméstica).

En cuanto a las sesiones, la intervención comenzará con una sesión de orientación, en la que se comunicará el propósito de la intervención en términos que tengan sentido para el participante, se proporcionará retroalimentación de la evaluación inicial y se explorarán empáticamente la experiencia migratoria, las dificultades actuales y las metas del participante. Luego, se presentarán brevemente al participante los componentes relevantes de la intervención, se eligirán los primordiales y se programarán en el calendario. Esto tiene como objetivo fomentar un ambiente de calidez, horizontalidad y cooperación, que los migrantes venezolanos han indicado en las entrevistas como culturalmente esperado.

Las siguientes 3 a 7 sesiones abordarán los síntomas más urgentes identificados y se discutirán con los participantes. Haciendo lugar a la esperanza, el guía comunicará al participante que los componentes presentados han demostrado ser eficaces para brindar alivio en personas con malestar similar. Los siguientes componentes se sugerirán en esta fase de la intervención:

- Re-estructuración cognitica: siempre
- Activación conductual: si el paciente tiene PHQ9 => 10 puntos
- Exposición a situaciones: si el paciente tiene GAD7 => 10 puntos
- Exposición a recuerdos: si el paciente tiene PCL5 => 10 puntos

Se realizará una variación de la intervención en función del malestar presente. En el caso de los participantes que puntúen por encima del punto de corte en los tres cuestionarios aplicados, se dedicarán más sesiones a las estrategias que abordan los síntomas. En el caso de los participantes con una sintomatología menos compleja, se dedicarán menos sesiones a esta. Una vez implementadas las estrategias anteriores, los participantes podrán optar por continuar con un conjunto complementario de sesiones (hasta alcanzar un total de 12 como máximo) que podrá incluir los siguientes componentes:

- Regulación emocional: Se sugerirá, si el participante describe dificultades para identificar, gestionar y expresar emociones (por ejemplo, arrebatos de ira, acumulación emocional y llanto repentino, impulsividad).
- Resolución de problemas: Se sugerirá, si el participante describe circunstancias concretas que le generan estrés y siente urgencia por cambiar (por ejemplo, desempleo, malas condiciones de vivienda, falta de apoyo con el cuidado de los niños, etc.) y si menciona sentirse estacando o paralizado con respecto a sus problemas, incapaz de generar un plan y abrumado por las decisiones que debería estar tomando.
- Foco en la adaptación: Se sugerirá, si el participante describe dificultades para sentirse "en casa" en el país anfitrión (por ejemplo, sentirse "fuera de lugar", miedo a perder la identidad venezolana, desconfiar y evitar a los peruanos, etc.).

Cada sesión vendrá con una tarea a poner en práctica en el tiempo entre sesiones. La última sesión destinará tiempo para revisar el proceso y elaborar un plan para seguir poniendo en práctica las estrategias aprendidas. También incluirá una

discusión sobre la posibilidad de recaída y las estrategias para prevenirla o recuperarse de ella.

A continuación, se describe brevemente el contenido de cada componente:

- Re-estructuración cognitiva: Se trata de una estrategia utilizada en el tratamiento de diferentes trastornos mentales. Introduce el triángulo “pensamiento-emoción-acción” y explora cómo estos tres aspectos se influyen entre sí. Los participantes examinan cómo un pensamiento puede afectar la forma en que se sienten y sus acciones. Aprenden a “optar” por pensamientos más útiles.
- Activación conductual: Se trata de una estrategia que se utiliza habitualmente en el tratamiento de la depresión. Introduce la idea de que la acción puede preceder a la motivación y no (solo) al revés. Se invita a los participantes a involucrarse en actividades placenteras, que se desglosan en pasos pequeños y factibles.
- Exposición a situaciones: Se trata de una estrategia utilizada en el tratamiento de distintas formas de ansiedad. Los participantes aprenden que la ansiedad suele conducir a conductas de evitación que la exacerban en una espiral de aislamiento. Al enfrentarse a las situaciones temidas (pero inofensivas) de forma gradual, aprenden que pueden superar los sentimientos de ansiedad y son capaces de involucrarse en más actividades.
- Exposición a recuerdos: Esta es una estrategia que se utiliza en el tratamiento del trastorno de estrés postraumático. A menudo existe la preocupación, de que se exacerben los síntomas, si se evoca intencionalmente los recuerdos que desencadenan malestar. Los participantes aprenden que es probable que la evitación de estos desencadenantes sea lo que mantiene los recuerdos intrusivos, lo cual es perjudicial. Evocan intencionalmente los recuerdos que provocan el malestar y vuelven a aprender que no son peligrosos, que pueden superar el malestar y que experimentarán menos malestar con el tiempo.
- Regulación emocional: Los participantes aprenden a identificar, nombrar y comunicar emociones incómodas. También examinan sus estrategias de afrontamiento cuando se sienten emocionalmente abrumados, las evalúan críticamente en términos de sus ventajas y desventajas y amplían su repertorio.
- Resolución de problemas: Los participantes aprenden un método para resolver problemas. Enumeran sus problemas más urgentes, eligen uno, lo describen en detalle, reúnen soluciones, seleccionan las estrategias más factibles y elaboran un plan de acción.
- Foco en la adaptación: Los participantes se centran en los sentimientos relacionados con la migración para poner en práctica las estrategias mencionadas anteriormente, pero esta vez con énfasis en apoyar su adaptación al país de acogida. Por ejemplo, los participantes utilizan la estrategia de “reestructuración cognitiva” para examinar cómo se sienten al haber migrado, identificar pensamientos comunes relacionados con esto y practicar formas nuevas y más útiles de pensar sobre el lugar en el que viven ahora. O los participantes utilizan la “activación conductual” para poner en práctica pequeños cambios que podrían mejorar la sensación placentera de “estar en casa” en sus “nuevos hogares”. O los participantes planifican una actividad con peruanos (por ejemplo, colegas, vecinos, padres de los amigos de sus hijos), si tienden a evitar pasar tiempo con peruanos (exposición a situaciones). O los participantes recuerdan las partes más dolorosas o angustiosas de la experiencia de haber migrado, como separarse de sus hijos, enfrentar dificultades durante el viaje o al llegar (exposición a recuerdos). En términos de regulación emocional, aprenden a oscilar entre sentimientos similares al duelo y un pensamiento centrado en el pasado y la implementación de estrategias de afrontamiento saludables y un pensamiento orientado al presente y al futuro.

i. Este proyecto se da en el marco de algún convenio o contrato, o posible financiación.
(X) Sí. Describirlo

Este proyecto se realiza gracias a una subvención del National Institute of Mental Health (Mecanismo K23: K23MH127308, https://grants.nih.gov/grants/guide/pa-files/PA-20-206.html, Mentored Patient-Oriented Research Career Development Award) otorgado a la Dra. Haley Carroll con la tutoría de los Dres. Bird y Freier.

( ) No.

ii. Señalar fecha probable de trabajo de campo: _________Enero 2025____________

iii. Proceso de reclutamiento de participantes
i) Describir el procedimiento para el reclutamiento de participantes. Tomar en cuenta:
( ) Si se requerirá alguna presentación formal por parte de la Universidad.
( ) Si se requerirá algún acuerdo o contrato con alguna institución.
( ) Si los sujetos de estudio son población vulnerable (menores de edad, sufren de alguna enfermedad o condición social especial).

Para el proceso de reclutamiento de participates, se recurrira2 al apoyo de varias instituciones: ONG activas en migración, postas médicas y centros comunitarios de salud mental de diferentes zonas de la ciudad, albergues de acogida que reciben migrantes, escuelas. Con su consentimiento, se realizarán las siguientes actividades de reclutamiento de participantes (opt-in):

- Se colocarán carteles en la fachada de estas organizaciones describiendo el estudio
- Se distribuirán volantes en estas instituciones
- Se repartirán volantes en eventos comunitarios organizados por ONG activas en migración
- Se publicarán avisos en las redes sociales de ONG activas en migración

Además, en coordinación con las ONG que ofrecen servicios de salud mental a migrantes venezolanos, se identificarán posibles sujetos elegibles, a quienes, debido a la falta de recursos, la ONG no pudo ofrecerles apoyo. Se pedirá a estas organizaciones que se pongan en contacto directo con estas personas para proporcionarles información sobre el estudio y darles la oportunidad de obtener más información mediante una llamada de seguimiento por parte del equipo de investigación o no (opt-out). En la llamada de seguimiento de parte de un miembro del equipo de investigación, se explicará el estudio. Si la persona está interesada en participar, se llevará a cabo la discusión de consentimiento para el tamizaje y luego el tamizaje. Eventualmente, les pediremos a los participantes que entreguen volantes a conocidos que puedan estar interesados en saber sobre el estudio y les comuniquen que pueden contactar directamente al equipo de investigación. Los carteles, volantes, publicaciones y comunicaciones verbales - inclusive el script para la llamada de reclutamiento - sobre el estudio incluirán información sobre el propósito del estudio, los criterios de elegibilidad, los compromisos, la modalidad en línea de la intervención y la información de contacto (ver apéndice).

ii) ¿Se realizará algún pago o se brindará algún otro tipo de incentivo para la participación?

(X) Sí. Señalar monto y procedimiento de pago:

Al final de la última sesión de intervención los participantes recibirán USD$ 5 o 20 PEN, por sesión de intervención (6 a 12 sesiones) y de evaluación (5 evaluaciones) a la que hayan asistido. Recibirá una compensación por la cantidad de sesiones que haya completado, incluso si se retira del estudio antes de concluir las sesiones. La compensación será en forma de un vale de consumo.<br><br>En el caso de los guías que implementen la intervención, recibirán USD$ 13 o 50 PEN, por cada sesión realizada. Los pagos se realizarán cada dos meses aproximadamente. Recibirán una compensación por la cantidad de sesiones que hayan realizado, incluso si se retiran del estudio antes de que llegue a su fin. Además, los guías recibirán una compensación por participar en el entrenamiento valorizada en aprox.1500 PEN por 10 días de entrenamiento.

( ) No.

II. Garantizar la participación voluntaria e informada de los sujetos de estudio (Consentimiento Informado).

i) Señalar los procedimientos que se emplearán para garantizar la participación voluntaria e informada de los participantes.

Dado que el objetivo principal del estudio es probar la eficacia de una intervención en el tratamiento de los trastornos mentales comunes (TMC), es un requisito previo para la participación que los individuos presenten síntomas de este tipo. Por lo tanto, es necesario un procedimiento de tamizaje, para el cual se prevee también un procedimiento de consentimiento que garatize la participación voluntaria e informada. Para esto se agendará una llamada con los posibles participantes que contacten al equipo de investigación o que consientan ser contactados por el equipo de investigación. En esta llamada, se describirá la intervención con mayor detalle (objetivo, duración, modalidad) y se llevará a cabo el procedimiento de consentimiento para el tamizaje. Se informará a los posibles participantes lo siguiente:<br>• Este es un estudio de investigación.<br>• Es necesario recopilar información para poder determinar si el estudio es adecuado para la persona.<br>• Un requisito previo es responder algunas preguntas sobre diferentes experiencias de malestar.<br>• Según las pruebas piloto de la evaluación, esperamos que el procedimiento de tamizaje no dure más de 30 minutos. Este se realice a través de una llamada telefónica.<br>• Una vez completados los cuestionarios, el equipo de investigación realizará una llamada de seguimiento. En función de las respuestas en las mediciones/puntuaciones, la llamada invitará al participante a participar en el proyecto de investigación, o en caso de que la persona no sea invitada, se le proporcionarán derivaciones a otras organizaciones que puedan ayudarles.<br>• Si la persona es invitada a participar del estudio, será asignada aleatoriamente para recibir tratamiento ahora o en 12 semanas.<br>• Se tomarán medidas para garantizar la confidencialidad y seguridad de los datos (se describirán las acciones específicas).<br>• Pueden decidir terminar su participación en el procedimiento de tamizaje en cualquier momento sin que ello les cause ninguna consecuencia negativa.

- Su participación en la selección no está vinculada a su participación en el estudio. A pesar de participar en la selección, pueden decidir no participar en el estudio.

Una vez que se haya entregado esta información y se hayan aclarado las preguntas, recopilaremos el consentimiento informado para la tamizaje mediante un procedimiento de consentimiento electrónico. Para ello, los participantes recibirán un enlace seguro a REDCap. Se adjunta el formato de consentimiento informado para el tamizaje.

Los participantes identificados en el tamizaje como elegibles en recibirán un mensaje invitándolos a participar del estudio. Dado que los participantes a los que se dirige este estudio son mayores de 18 años, ellos mismos darán su consentimiento en un procedimiento electrónico de consentimiento informado, para lo cual se programará una llamada. Durante la llamada, un miembro capacitado del equipo de investigación describirá el estudio en términos no técnicos en español. El personal del estudio recibirá información sobre los procedimientos necesarios para obtener el consentimiento informado, y se repetirá la capacitación y la supervisión para reforzar continuamente estos procedimientos. Todo el personal del estudio también recibirá capacitación sobre conducta ética de la investigación con sujetos humanos y deberá re-certificarse anualmente.

A todas las personas se les informará que pueden retirar su consentimiento para participar en cualquier momento durante el estudio, sin ninguna consecuencia. Se informará a los participantes potenciales que la información proporcionada a través del consentimiento y en las sesiones es confidencial (es decir, no se compartirá con nadie fuera del equipo de investigación) y voluntaria (es decir, no están obligados a compartirla). El miembro del personal de investigación enfatizará que la participación en el estudio es completamente voluntaria, que los participantes pueden retirar su consentimiento y abandonar el estudio en cualquier momento. El personal de investigación recibirá una capacitación integral para garantizar que los participantes den su consentimiento informado de manera voluntaria. Si en algún momento el participante potencial no está interesado en el estudio, el miembro del personal de investigación le agradecerá por tomarse el tiempo para aprender sobre el estudio y luego registrará la información sobre por qué no estaba interesado en participar.

El formulario de consentimiento informado proporcionará la siguiente información de manera integral: (a) introducción al proceso de consentimiento, explicando el formulario de consentimiento y el cumplimiento de la política institucional y las leyes del país; (b) recordatorio de que la participación es voluntaria; (c) naturaleza y propósito del estudio; (d) explicación de los procedimientos del estudio; (e) posibles incomodidades y riesgos, así como planes para proteger a los participantes de estos riesgos; (f) posibles beneficios; (g) confidencialidad, incluyendo cómo se utilizarán los datos y cómo se mantendrán privados; (h) rechazo/retiro, incluyendo el derecho a retirar el consentimiento y abandonar el estudio en cualquier momento; y (i) derechos y quejas. El miembro del equipo de investigación leerá un resumen de cada sección al participante. Después de cada sección principal, el miembro del equipo de investigación hará una pausa y comprobará la comprensión, por ejemplo, pidiendo al participante que repita, con sus propias palabras, qué significa "el derecho a negarse".

Una vez que se hayan aclarado las preguntas y se haya compartido toda la información, se invitará a los participantes a dar su consentimiento electrónicamente a través de un enlace en REDCap. En REDCap, los participantes encontrarán el formulario de consentimiento, una afirmación de que entienden el estudio y consienten participar, un recuadro para ingresar su nombre y un recuadro para ingresar la fecha. Además, encontrarán dos casillas para marcar, en caso de que den su permiso para ser contactados nuevamente por el equipo de investigación con el propósito de este estudio y para otros estudios. Se les recordará que pueden retirarse en cualquier momento y se les proporcionará la información de contacto para hacerlo. Después de la llamada, los participantes recibirán una copia en PDF del formulario de consentimiento firmado a través de WhatsApp.

Dado que se recogerá datos de los guías para medir la viabilidad y aceptabilidad de la intervención, estos también pasarán por un procedimiento de consentimiento informado. Se adjunta el formato correspondiente.

ii) Adjuntar Consentimiento Informado que se planea utilizar en el estudio
Se adjuntan:

- Formato de consentimiento informado para el proceso de tamizaje
- Formato de consentimiento informado para participar del estudio como sujeto
- Formato de consentimiento informado para participar del estudio como guía.

III. Gestión de la información sensible de los participantes

i) Durante la aplicación del campo se recogerán datos personales de los individuos como (marque los que se recogerán):
(X) Nombres
( ) Iniciales
(X) Direcciones o códigos postales
(X) Teléfonos o correos
(X) Número de documento de identidad
( ) Fotografías de los participantes
(X) Datos de parientes.
( ) Ninguno (si respondió esta alternativa, pase a la pregunta iii)

ii) Si va a recoger algún dato señalado en la pregunta anterior, señale si se realizará algún procedimiento para anonimizar estos datos.
- Sí, se procederá con anonimizar los datos. Describa cómo lo realizará:

A los participantes se les asignará un ID único de estudio, asociándose los datos del estudio únicamente a estos ID. Los participantes serán identificados en todos los materiales del estudio solo por el número de participante, el número de sesión y la fecha de la sesión. Se guardará una lista maestra de los nombres de los participantes y sus identificaciones del estudio en un archivador cerrado con llave y separado del resto de los datos. Los doctores, Carroll, Freier y Bird, coordinadores de la investigación del estudio, así como Talía Guevara, miembro del equipo de la investigación, serán las únicas personas con acceso a esta lista. La lista maestra se destruirá al finalizar el estudio. En las transcripciones para evaluar la fidelidad de la implementación se utilizarán los números de los participantes, no sus nombres. Al registrar los datos del estudio de esta manera, la información puede considerarse "no identificada" y, por lo tanto, cumple con los Estándares de Privacidad de la Información sanitaria individualmente identificable (Reglas de Privacidad) de la

HIPAA.

- No. Señale por qué no realizará este proceso.

iii) Señale quién será el encargado de resguardar la información recogida.

La persona encargada de salvaguardar la información y supervisar que así se ejecute será la Dra. Haley Carroll, líder del equipo de investigación que llevará a cabo este proyecto.

iv) ¿Dónde se almacenará la información recogida y si se empleará algún método para resguardar su acceso?

Los datos sobre elegibilidad, el tamizaje de los participantes, información demográfica, mediciones de resultados (PHQ-9, GAD-7, LEC-5 y PCL-5), potenciales variables moderadoras (IPL), adherencia y aceptabilidad se ingresarán directamente en el sistema electrónico REDCap, software seguro protegido por constraseña y encriptado. Los datos sobre viabilidad (reclutamiento y retención) se ingresarán en una hoja de Excel.

Los formularios de datos pasarán por un proceso de edición sistemático y riguroso antes de ser ingresados en la base de datos. El coordinador de la investigación (Talía Guevara, Universidad del Pacífico) y el investigador principal (PI) evaluarán rutinariamente los datos y discutirán cualquier problema o duda con el equipo de investigación en las reuniones regulares semanales. Se emplearán informes de gestión de datos en las tres áreas siguientes: ingresados, verificados y editados. Estos informes de registros de datos serán evaluados una vez al mes. Para ayudar a garantizar la protección de los datos, se generarán copias de seguridad cada semana. Además, nuestros sistemas de registros en formato físico, como se describió previamente, se mantendrán en gabinetes a prueba de fuego y con llave en cada sitio. Los datos recopilados en Perú de las evaluaciones del estudio estarán disponibles para el PI y el personal de investigación en REDCap. Además, los datos sobre la capacitación, la supervisión y el progreso de los participantes también se ingresarán y cargarán. Esta entrada de datos ocurrirá lo más cerca posible del tiempo real para facilitar la gestión de los datos y el monitoreo de las operaciones del estudio.

Todas las grabaciones de audio, las notas y las evaluaciones cuantitativas completadas se guardarán en un archivador cerrado con llave y se mantendrán en zonas restringidas. Todas las memorias USB y los ordenadores estarán encriptados y protegidos por contraseña. Las versiones electrónicas de las grabaciones de audio, las notas y los datos cuantitativos estarán protegidos por contraseña. Las contraseñas cambiarán periódicamente y sólo serán accesibles para el personal de investigación especificado. Todas las grabaciones de audio digitales se cargarán en el ordenador del estudio inmediatamente después de la grabación y el archivo de audio se eliminará de la grabadora digital.

Los registros del estudio, los formularios de consentimiento para la

selección y para la participación, así como los datos recopilados durante el estudio, se conservarán durante 7 años después de que el estudio se cierre y luego serán destruidos. Estos se preservarán en formato electrónico.

IV. Posibles riesgos del estudio

i) Como parte del estudio, en algún momento los participantes deberán responder a preguntas sobre temas sensibles (sexualidad, política, etc.):
   (X) Sí.
   ( ) No.

ii) Como parte del estudio, los participantes deberán realizar alguna acción que pueda poner en riesgo su integridad física o mental.
   (X) Sí.
   ( ) No.

iii) Como parte del estudio, los investigadores del estudio deberán realizar alguna acción que pueda poner en riesgo su integridad física o mental.
   ( ) Sí.
   (X) No.

iv) En caso de haber contestado "Sí" a alguna de estas preguntas, describir en qué consisten estas acciones y exponer las razones de la necesidad de estas acciones.

Es poco probable que los participantes corran riesgo de sufrir daño físico como resultado de la participación en el estudio. Los principales riesgos para los participantes son la violación de la confidencialidad, la fatiga o el malestar personal durante las sesiones de intervención o las evaluaciones. Los participantes pueden encontrar algunos de los temas abordados durante las sesiones o algunas de las preguntas formuladas durante las evaluaciones emocionalmente perturbadoras y pueden experimentar aumentos a corto plazo de emociones negativas durante las sesiones de intervención. Como en cualquier estudio de participantes con malestar psicológico, siempre existe el riesgo de que los síntomas empeoren. La evaluación semanal de los síntomas durante la intervención permitirá monitorear de cerca cualquier cambio y poner en marcha un plan de seguridad, en caso de aumento de la gravedad o tendencia suicida. Las reuniones semanales con los supervisores, acompañados de cerca por el equipo de investigación en reuniones grupales semanales, permitirán identificar oportunamente a los participantes cuyos síntomas no están progresando como se esperaba y tomar medidas oportunas. En cualquier momento del estudio, si los participantes presentan una gravedad creciente de los síntomas psiquiátricos que justifica un mayor nivel de atención, serán remitidos al hospital local. Si los síntomas requieren tratamiento médico inmediato, se contactará a los familiares de los participantes y se les recomendará llevarlos a la sala de emergencias del hospital. Además, se recomendará a los participantes llamar a la línea telefónica 113 para ponerse en contacto con consejeros en salud mental del MINSA (Ministerio de Salud) o al 106, si necesitan una ambulancia.

En el caso de los participantes guía, provedores de la intervención, es posible que algunos de los temas tratados durante las sesiones les resulten emocionalmente perturbadores o que experimenten preocupación por el bienestar de algunos participantes. Como se mencionó anteriormente, las reuniones frecuentes con los supervisores, acompañadas de cerca por el equipo de investigación, permitirán discutir estas preocupaciones, identificar si los síntomas de un participante no están progresando como se esperaba y tomar medidas oportunas. Además, las supervisiones ofrecerán la

oportunidad de abordar el malestar que un proveedor pueda estar experimentando y encontrar maneras de aliviarlo.

Otras medidas que tomaremos para proteger de riesgos a los participantes son las siguientes:

- Equipo de investigadores experimentados, con amplia experiencia en desplazamiento forzado y salud mental
- Capacitación rigurosa en conducción a ética de investigación humana para todo el personal y capacitación relacionada con el proyecto sobre el trauma relacionado con la migración y la comprensión de la salud mental.
- Medidas para garantizar la privacidad y la confidencialidad, como asegurarse de que no participen otras personas en las sesiones, mantener los documentos y la información en entornos seguros y cerrados, identificar a los sujetos individuales por número de estudio sin identificadores personales
- Ofrecer descansos para aliviar la fatiga y el malestar; recordatorios de la voluntariedad de su participación en el estudio
- Plan de derivación en caso de riesgo de daño, depresión grave y tendencias suicidas
- Volver a experimentar el trauma es una preocupación común, pero se ha demostrado que el proceso de exposición es eficaz en cientos de ensayos clínicos para el TEPT[7]. La capacitación y la supervisión de los guías garantizarán que se comprenda la lógica, se respete la estructura y se desarrollen las habilidades para ayudar a los participantes a interactuar adecuadamente con los estímulos traumáticos.

Dicho esto, es importante mencionar que esperamos ver una disminución de los síntomas relacionados con la depresión, la ansiedad y el TEPT a lo largo del tiempo de la intervención. Por lo tanto, el principal beneficio potencial para los sujetos es una mejora en su salud mental. Además, dado que la intervención contempla componentes de “resolución de problemas” y “regulación emocional”, los beneficios previsibles serían mejoras en las situaciones que los participantes identifican como problemáticas, así como en su capacidad para gestionar las emociones estresantes. Además, dado que la intervención aborda aspectos específicos relacionados con la migración, como el “duelo migratorio”, un beneficio potencial es una mejora en la integración psicológica al país de acogida.

Además, esperamos que el estudio proporcione conocimiento sobre la eficacia de una intervención específicamente diseñada para abordar el trastornos mentales comunes en la población de migrantes venezolanos. Hasta el momento no se ha generado evidencia sobre la eficacia de intervenciones de trastornos mentales comunes que sean culturalmente aceptables y contextualmente factibles para aliviar la ansiedad, la depresión y el TEPT entre los migrantes venezolanos que viven en Perú, que ha recibido más de 1.5 millones de migrantes de Venezuela. En términos más generales, esperamos que el estudio propuesto brinde conocimiento sobre la viabilidad y eficacia de un protocolo de tratamiento transdiagnóstico para abordar los trastornos de salud mental en poblaciones desplazadas por la fuerza que han migrado a entornos con recursos limitados. Se estima que la prevalencia

[7] Cuijpers P, Berking M, Dobson KS, Andersson G, Quigley L, Kleiboer A. A Meta- Analysis of Cognitive-Behavioural Therapy for Adult Depression, Alone and in Comparison with other Treatment. *The Canadian Journal of Psychiatry*. 2013;58(7). doi:https://doi.org/10.1177/07067437130580070

global del desplazamiento forzoso es de 70 millones de personas por año. Aunque es difícil evaluar con precisión la carga exacta de enfermedades en las poblaciones desplazadas forzosamente, los cálculos sobre la prevalencia de trastornos mentales varían entre aproximadamente el 10% y el 30% para la depresión, la ansiedad y el TEPT.

En el caso de los participantes proveedores de la intervención, esperamos que la capacitación aporte al desarrollo de capacidades que puedan serles de utilidad en oportunidades futuras.

La población a la que pertenecen los sujetos del estudio tiene un acceso muy limitado a los servicios de salud mental. Debido a las barreras burocráticas, las actitudes xenófobas y la falta de recursos, los migrantes venezolanos en su mayoría quedan fuera del sistema de salud mental provisto por el Estado peruano. Además, la disminución de la disponibilidad de fondos está poniendo en peligro la continuidad de los servicios de salud mental brindados por las ONG que priorizan la migración, como se ha informado en grupos focales con expertos. Por lo tanto, las intervenciones de salud mental específicamente diseñadas para esta población, que estén orientadas a la gente común y fortalezcan los recursos a nivel comunitario y de atención primaria, son prometedoras. Con este estudio esperamos contribuir a ampliar la cobertura de salud mental. Además, en relación con los participantes del estudio, se espera ofrecer una intervención que aborde y alivie su malestar en un contexto donde, de otro modo, quedaría desatendido debido a las restricciones mencionadas anteriormente. Aunque el malestar podría aumentar inicialmente, la intervención se basa en protocolos de tratamiento bien respaldados, que han demostrado su eficacia en numerosos estudios en diferentes poblaciones. Por lo tanto, se espera contribuir en general al bienestar de los sujetos.

V. Instrumentos y aplicación de trabajo de campo

i) Describa cómo se realizará el trabajo de campo. Señalar si se realizará de manera presencial u online, el tiempo de intervención, escenarios, etc.

<u>Reclutamiento:</u>

Opt-in: Los participantes potenciales se informan sobre el estudio y se ponen en contacto con el equipo de investigación. La información se comparte a través de

- Afiches en las fachadas de las ONG activas en migración, centros de salud y centros comunitarios de salud mental, albergues, escuelas
- Volantes disponibles en estas instituciones
- Volantes distribuidos en eventos comunitarios organizados por ONG activas en migración
- Se publicarán avisos en las redes sociales de las ONG activas en migración

Opt-out: Las ONG que ofrecen servicios de salud mental a migrantes venezolanos identifican a posibles sujetos elegibles, a quienes, debido a la falta de recursos, la ONG no pudo ofrecerles apoyo. Las organizaciones se comunican directamente con estas personas para brindarles información sobre el estudio y darles la oportunidad de obtener más información mediante una llamada de seguimiento por parte del equipo de investigación o no.

<u>Consentimiento para el tamizaje:</u>

Se programará una llamada para presentar la información sobre el procedimiento de evaluación, aclarar dudas y verificar la comprensión. El consentimiento electrónico se recogerá a través de REDCap.

Evaluación de tamizaje y línea de base:
Se realizará online a través de un link seguro a REDCap. Se aplicarán los cuestionarios PHQ9, GAD7, PCL5, LEC5, cuestionario demográfico, cuestionario con criterios de exclusión.
Consentimiento de participación y enlistamiento:
Un link al formulario de consentimiento para la participación se enviará por WhatsApp a los sujetos que califican. Se programará una llamada para presentar la información sobre el procedimiento de evaluación, aclarar dudas y verificar la comprensión. El consentimiento electrónico se recogerá a través de REDCap.
Randomización y comunicación del inicio de la intervención:
Los resultados de la asignación aleatoria a "intervención inmediata" o "grupo control de lista de espera", a realizarse por un generador de números, se comunicará via una llamada telefónica. El formato de información demográfica completo y el cuestionario IPL se enviarán por un link seguro de REDCap.
Sesión 1 (sesión de orientación):
Se implementará online vía Meet or WhatsApp, individualmente con el guía. Tendrá 60 minutos de duración. Se enfocará en conocerse, plantearse metas, agendar las sesiones, ponerse de acuerdo en metas para la cancelación y postergación de citas.
Sesiones 2 a 6:
Se implementarán online vía Meet or WhatsApp, individualmente con el guía. Tendrán 60 minutos de duración. Se enfocarán en el alivio de síntomas (depresión, ansiedad, estrés post-traumático).
Sesiones 7 a 12:
Se implementarán online vía Meet or WhatsApp, individualmente con el guía. Tendrán 60 minutos de duración. Se enfocarán en otros aspectos relevantes para la salud mental (regulación emocional, resolución de problemas, malestar relacionado con la adaptación).
Evaluaciones durante la intervención:
Se realizarán online mediante un link seguro a REDCap (PCL5 cada semana antes de cada sesión, PHQ9 y GAD7 cada dos semanas antes de la sesión, aceptabilidad y adherencia/fidelidad cada semana después de cada sesión).
Línea final y evaluaciones de seguimiento al mes, a los 3 meses y a los 6 meses:
Se realizarán online mediante un link seguro a REDCap (PHQ9, GAD7, LEC5, PCL5) 12 semanas después de la primera sesión de intervención o 12 semanas después de la línea de base en el caso del grupo control en lista de espera y 1 mes, 3 meses y 6 meses después de la línea final.
Análisis de datos:
El análisis de datos se realizará mediante software de análisis de datos y se utilizará para su publicación y difusión en revistas académicas y conferencias.
Monitoreo y reporte de seguridad:
Evento Adverso (EA): es cualquier resultado negativo que sea indeseable e involuntario, derivado de la participación de un sujeto en un estudio. Estos eventos pueden abarcar diversas formas de daño, incluyendo impactos físicos, psicológicos, sociales, legales o económicos. Por ejemplo, en la investigación social y conductual, un ejemplo de evento adverso podría ser una violación de la confidencialidad, donde la información privada de un participante se divulga a personas no involucradas en el equipo de investigación.

Problema No Anticipado (PNA): Se define como un evento adverso que cumple con tres criterios específicos:
1. Naturaleza inesperada: El evento adverso debe ser inesperado, es decir, su naturaleza, gravedad o frecuencia no debe coincidir con los riesgos

conocidos o previsibles descritos en el protocolo de investigación, los documentos de consentimiento informado u otras fuentes relevantes.

2. Relación con la investigación: El evento adverso debe estar relacionado o posiblemente relacionado con la participación en la investigación. Esto significa que existe una posibilidad razonable de que el evento haya sido causado por los procedimientos de investigación.
3. Aumento del riesgo de daño: El evento adverso debe sugerir que la investigación coloca a los sujetos u otras personas en un mayor riesgo de daño del que se conocía previamente. Esta evaluación implica considerar si el evento indica un riesgo nuevo que no se había anticipado según el conocimiento previo de la investigación y sus procedimientos.

Evento Adverso Grave (EAG): cualquier evento adverso que

1. Resulta en la muerte;
2. Es potencialmente mortal;
3. Resulta en hospitalización o prolongación de una hospitalización existente;
4. Resulta en una discapacidad/incapacidad persistente o significativa;
5. Resulta en una anomalía congénita/malformación al nacer; o abuso.

Potencialmente mortal significa que el evento coloca al sujeto en riesgo inmediato de muerte debido al evento tal como ocurrió.

Los procedimientos para proteger a los participantes se describen a continuación:

1. Protocolos de Comunicación Regular
   - Reuniones Semanales del Equipo: El investigador principal (PI) y el coordinador de investigación participarán en reuniones semanales regulares para discutir la seguridad continua de los participantes, revisar los eventos reportados y abordar cualquier inquietud.
   - Revisión Mensual de Datos: Se llevará a cabo una revisión sistemática de los datos cada mes, en la que el equipo de investigación evaluará la frecuencia y naturaleza de los eventos adversos y eventos adversos graves reportados.
2. Comunicación entre Sitios
   - Notificación Inmediata de EAGs y PNAs: Cualquier miembro del equipo de investigación que identifique un evento adverso grave o un problema no anticipado notificará al PI dentro de las 24 horas posteriores a la identificación del evento. Esto asegura que todos los sitios estén informados de los posibles riesgos y puedan ajustar sus prácticas de monitoreo de manera adecuada.
   - Informe al IRB: El PI se asegurará de que todos los PNAs y EAGs sean reportados a los Comités de Revisión Institucional (IRB) correspondientes (BMC y IRB de la Universidad del Pacífico) dentro del plazo requerido, y se proporcionarán actualizaciones a todos los sitios.
3. Modificaciones del Protocolo
   - Enmiendas al Protocolo: Si se requieren modificaciones al protocolo basadas en los resultados interinos o en el monitoreo de seguridad, estas se comunicarán de inmediato a todos los sitios. Los sitios afectados recibirán instrucciones detalladas sobre cómo implementar los cambios y se les pedirá confirmar su comprensión y cumplimiento.
4. Capacitación y Cumplimiento
   - Capacitación del Personal del Sitio: Todo el personal de los sitios recibirá capacitación sobre los procedimientos de monitoreo de seguridad y los protocolos de comunicación establecidos para el estudio. Esta capacitación garantizará que cada miembro del equipo entienda su

rol en la seguridad de los participantes y la importancia de realizar informes oportunos.

Plan de reporte:

Si ocurren eventos adversos (es decir, eventos que son perjudiciales para los participantes del estudio), el personal será capacitado para completar las descripciones de los eventos adversos, las cuales serán transmitidas electrónicamente al Dr. Carroll dentro de las 24 horas. Si el evento es un Problema No Anticipado (PNA), se reportará al IRB a través del sistema electrónico del IRB del BMC (Boston Medical Center). Dado que este es un estudio multisitio, el Investigador Principal (PI) reportará cualquier PNA interno y externo a los IRBs locales y al IRB principal, siguiendo las directrices requeridas. Los informes se enviarán tan pronto como sea posible, pero no más tarde de 7 días después de que el investigador o el personal de investigación se entere del incidente. Cada informe describirá el PNA y especificará cualquier cambio requerido en la investigación o explicará por qué no son necesarios cambios. Si se requieren cambios, se enviará una solicitud de enmienda separada, pero esto no debe retrasar el informe inicial.

Además, el Investigador Principal enviará un informe resumen de todos los Eventos Adversos (EA), incluidos los Eventos Adversos Graves (EAG) que NO se clasifiquen como PNA, al IRB a través del sistema electrónico del IRB del BMC (Boston Medical Center) durante la revisión continua y al momento del cierre del estudio. Un informe formal de un monitor de seguridad de datos cumplirá con el requisito de este informe resumen. El resumen incluirá un análisis que evalúe si el patrón general de eventos indica que la investigación presenta un mayor riesgo de daño para los sujetos u otros, considerando el daño físico, psicológico, económico o social en función de la naturaleza y frecuencia de los eventos.

Al momento de la inscripción, se recordará a los participantes que el formulario de consentimiento informado contiene información sobre cómo contactar al coordinador de investigación y al Investigador Principal en el sitio del estudio para reportar cualquier evento adverso o cualquier pregunta o inquietud relacionada con el estudio.

Se llevará a cabo una revisión expedita para todos los eventos que cumplan con la definición de Eventos Adversos Graves (EAGs) de la Oficina para la Participación Humana en la Investigación (OHRP, por sus siglas en inglés) de 2007 (es decir, cualquier evento fatal, evento inmediatamente mortal, que requiera hospitalización como paciente interno, evento discapacitante de forma permanente o sustancial, que ponga en peligro la salud o cualquier anomalía congénita). Esto también incluye cualquier evento que un investigador del estudio considere que impone un peligro significativo, contraindicación, efecto secundario o precaución.

Para los fines de este estudio, todos los EAGs deberán ser reportados al IRB del BMC (Boston Medical Center) y a los mentores de investigación, los Drs. Feline Freier y Matthew Bird, y a la Universidad del Pacífico, independientemente de su relación con el estudio. Se reportará toda la información pertinente, incluida información sobre el evento y su resultado, los medicamentos concomitantes, el historial médico del sujeto y las condiciones actuales, y todos los datos de laboratorio pertinentes. Como se mencionó anteriormente, los informes de EAGs

se enviarán tan pronto como sea posible, pero no más tarde de 7 días después de que el investigador o el personal de investigación se entere del incidente. La información será revisada, y se determinará si existe alguna posible relevancia para las intervenciones del estudio. Sin embargo, ni la Universidad del Pacífico ni los investigadores que la representan asumen ningún tipo de costo o compensación que pueda surgir. La participación es voluntaria e informada (el participante puede retirarse en cualquier momento) y, como tal, no es conveniente asumir ningún coste por tratamiento, atención, asesoramiento o daño.

Reglas de interrupción:
Si en cualquier momento durante el estudio, los investigadores, mentores o los Comités de Revisión Institucional (IRB, por sus siglas en inglés) consideran que el riesgo para los sujetos supera los posibles beneficios, tendrán la discreción y responsabilidad de recomendar que el estudio sea terminado. Sin embargo, no tenemos una regla de interrupción pre-establecida.

ii) Adjunte los instrumentos a emplear, sean protocolos de entrevistas, encuestas u otros materiales tal cual se presentarán al participante.
Adjunto (ver apéndice).

iii) ¿Se realizarán grabaciones o algún otro tipo de registro de audio o video de los participantes?
(X) Sí. Señalar lo que se empleará y si esto estará consignado en el Consentimiento Informado.

Las sesiones de intervención serán grabadas en audio con el permiso del participante dentro de los procedimientos de consentimiento informado. Un 20% de las grabaciones de las sesiones será seleccionado para el análisis de la fidelidad y calidad de la intervención. Estas grabaciones de audio serán transcritas literalmente y traducidas. Se aplicará una ficha de adherencia a las transcripciones (ver adjunto). Los resultados de esta evaluación servirán de base para la discusión grupal y el feedback a los guías.

( ) No.

iv) ¿Durante la aplicación se emplearán algún tipo de logo o identidad gráfica de la Universidad del Pacífico?
(X) Sí. Describir y adjuntar, de ser necesario, cuáles.
( ) No.

Ver modelo de afiche adjunto.

VI. Posibles conflictos de interés

i) De acuerdo con el Código de Ética de la Universidad, posibles conflictos de interés pueden suscitarse de las siguientes situaciones:
- Cuando el investigador o sus familiares cercanos tengan un interés financiero en alguna entidad que auspicie la investigación o tengan un interés económico directo en el resultado, o cuando de otra forma puedan beneficiarse personalmente de ella.
- Cuando se proponga la contratación de familiares cercanos del investigador en el proyecto, sin que hayan pasado por un proceso de selección transparente.

- Cuando haya un conflicto de compromiso entre las actividades externas del investigador y su responsabilidad como trabajador de la Universidad. Los profesores-investigadores de la Universidad deben dedicar su tiempo y brindar sus servicios profesionales de manera prioritaria a esta casa de estudios.
- Cuando se pueda comprometer a la Universidad en el respaldo o la aprobación de alguna empresa, producto comercial, proyecto de ley o postura partidaria.
- Cuando el investigador, cuya principal afiliación sea con la Universidad, también tenga afiliaciones con entidades del sector privado empresarial o del gobierno, y a la vez aparezca en los medios de comunicación como experto, sin divulgar claramente estas afiliaciones.

ii) Si usted considera que podría existir algún conflicto de interés en su estudio por los motivos señalados anteriormente u otros, por favor indíquelos a continuación. En caso de que no los hubiera, señálelo también:

No hay conflictos de interés.
